# Cytotoxic activity of triazole-containing alkyl β-D-glucopyranosides on a human T-cell leukemia cell line

**DOI:** 10.1186/s13065-014-0072-1

**Published:** 2015-02-01

**Authors:** Edward Davis Oldham, Larissa M Nunes, Armando Varela-Ramirez, Stephen E Rankin, Barbara L Knutson, Renato J Aguilera, Hans-Joachim Lehmler

**Affiliations:** Department of Chemistry, University of Mary Washington, 1300 College Avenue, Fredericksburg, VA 22401 USA; Cytometry, Screening and Imaging Core Facility, Border Biomedical Research Center, Department of Biological Sciences, Bioscience Research Building, University of Texas at El Paso, 500 West University Ave., El Paso, TX 79968 USA; Department of Chemical and Materials Engineering, University of Kentucky, Lexington, KY 40506 USA; Department of Occupational and Environmental Health, The University of Iowa, UI Research Park, Iowa City, IA 52242 USA

## Abstract

**Background:**

Simple glycoside surfactants represent a class of chemicals that are produced from renewable raw materials. They are considered to be environmentally safe and, therefore, are increasingly used as pharmaceuticals, detergents, and personal care products. Although they display low to moderate toxicity in cells in culture, the underlying mechanisms of surfactant-mediated cytotoxicity are poorly investigated.

**Results:**

We synthesized a series of triazole-linked (fluoro)alkyl β-glucopyranosides using the copper-catalyzed azide-alkyne reaction, one of many popular “click” reactions that enable efficient preparation of structurally diverse compounds, and investigate the toxicity of this novel class of surfactant in the Jurkat cell line. Similar to other carbohydrate surfactants, the cytotoxicity of the triazole-linked alkyl β-glucopyranosides was low, with IC_50_ values decreasing from 1198 to 24 μM as the hydrophobic tail length increased from 8 to 16 carbons. The two alkyl β-glucopyranosides with the longest hydrophobic tails caused apoptosis by mechanisms involving mitochondrial depolarization and caspase-3 activation.

**Conclusions:**

Triazole-linked, glucose-based surfactants **4a-g** and other carbohydrate surfactants may cause apoptosis, and not necrosis, at low micromolar concentrations via induction of the intrinsic apoptotic cascade; however, additional studies are needed to fully explore the molecular mechanisms of their toxicity.

**Graphical Abstract:**
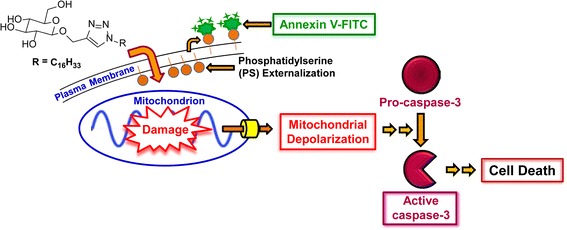
Triazole-linked, glucose-based surfactants cause apoptosis, and not necrosis, at low micromolar concentrations via induction of the intrinsic apoptotic cascade.

**Electronic supplementary material:**

The online version of this article (doi:10.1186/s13065-014-0072-1) contains supplementary material, which is available to authorized users.

## Background

Carbohydrate surfactants are an important class of surfactants that can be produced from renewable raw materials (e.g., starch, cellulose, hemicellulose, etc.) and are considered to be environmentally safe. Because of their interesting interfacial properties, carbohydrate surfactants with hydrocarbon tails are useful for a broad range of applications, such as pharmaceuticals, detergents, agrochemicals, food and personal care products [1-3]. Carbohydrate surfactants with partially fluorinated tails are of particular interest for biomedical applications, including blood substitutes and pulmonary drug delivery [4-7]. One feature of carbohydrate surfactants is the availability of an incredible number of structural motifs, including varied head groups, hydrophobic tails and linkers [2]. For example, the polar head group of carbohydrate surfactants can contain one or more mono- to polysaccharide moieties; be cyclic or linear; or differ in the stereochemistry of the hydroxyl groups. Furthermore, the carbohydrate head group can be linked by a variety of approaches, for example glycosylation, esterification and etherification, and using different linkers to one or more hydrophobic tails.

The copper-catalyzed azide-alkyne cycloaddition (CuAAC) [8] between an azide and an alkyne represents an attractive and straightforward approach to link a polar carbohydrate headgroup and a hydrophobic tail. Indeed, a considerable number of carbohydrate surfactants containing a 1,2,3-triazole linker have been described, including simple alkyl xylopyranoside [9] and glucopyranoside surfactants [10], structurally more complex glucose and maltose-based conjugates [11-14], alkyl and aryl *O*-xylosides and *O*-xylobiosides [15], 6-triazole-linked galacto- or glucolipids [16], branched fluorinated amphiphiles [17], bolaform surfactants with glucose, galactose and lactose head groups [18], mannitol-based gemini surfactants [19], and “star-like” carbohydrate surfactants [20]. Many triazole-linked carbohydrate surfactants can be synthesized by the reaction of a carbohydrate group containing an azide group with a suitable alkyne derivative, such as alkynes [18] or propargyl derivatives of alcohols [12,14] and fatty acids [11,13,16]. Alternatively, a carbohydrate group with a propargyl group can be reacted with alkyl azides to yield the desired triazole-linked carbohydrate surfactants [9,10,15,18,19].

The synthesis and physicochemical properties of carbohydrate surfactants have been investigated in considerable depths [3,21,22]. However, only limited structure-toxicity relationships of carbohydrate surfactants in general and triazole-liked carbohydrate surfactants in particular have been reported in mammalian systems. Typically, carbohydrate surfactants display low toxicity in cells in culture, with IC_50_ values in the micro- to millimolar concentration range [4-7,9,13,23-29]. For example, we observed IC_50_ values ranging from 26 to 890 μM for a series of triazole-linked alkyl β-D-xylopyranosides in several mammalian cell lines, with the Jurkat cell line being the most sensitive cell line [9]. Despite the potential use of carbohydrate surfactants in food and personal care products and biomedical applications, the mechanisms underlying the cytotoxicity of carbohydrate surfactants have not been explored systematically to date. Because of the potentially broad application of the triazole-linker in the synthesis of structurally diverse carbohydrate surfactants, we prepared a series of triazole-linked alkyl β-D-glucopyranosides with hydrocarbon and partially fluorinated hydrophobic tails, and performed a preliminary investigation of possible mechanisms of their toxicity in comparison to other carbohydrate surfactants in the Jurkat cell line.

## Results and discussion

### Synthesis of triazole-linked alkyl glucopyranosides

**Scheme 1:**
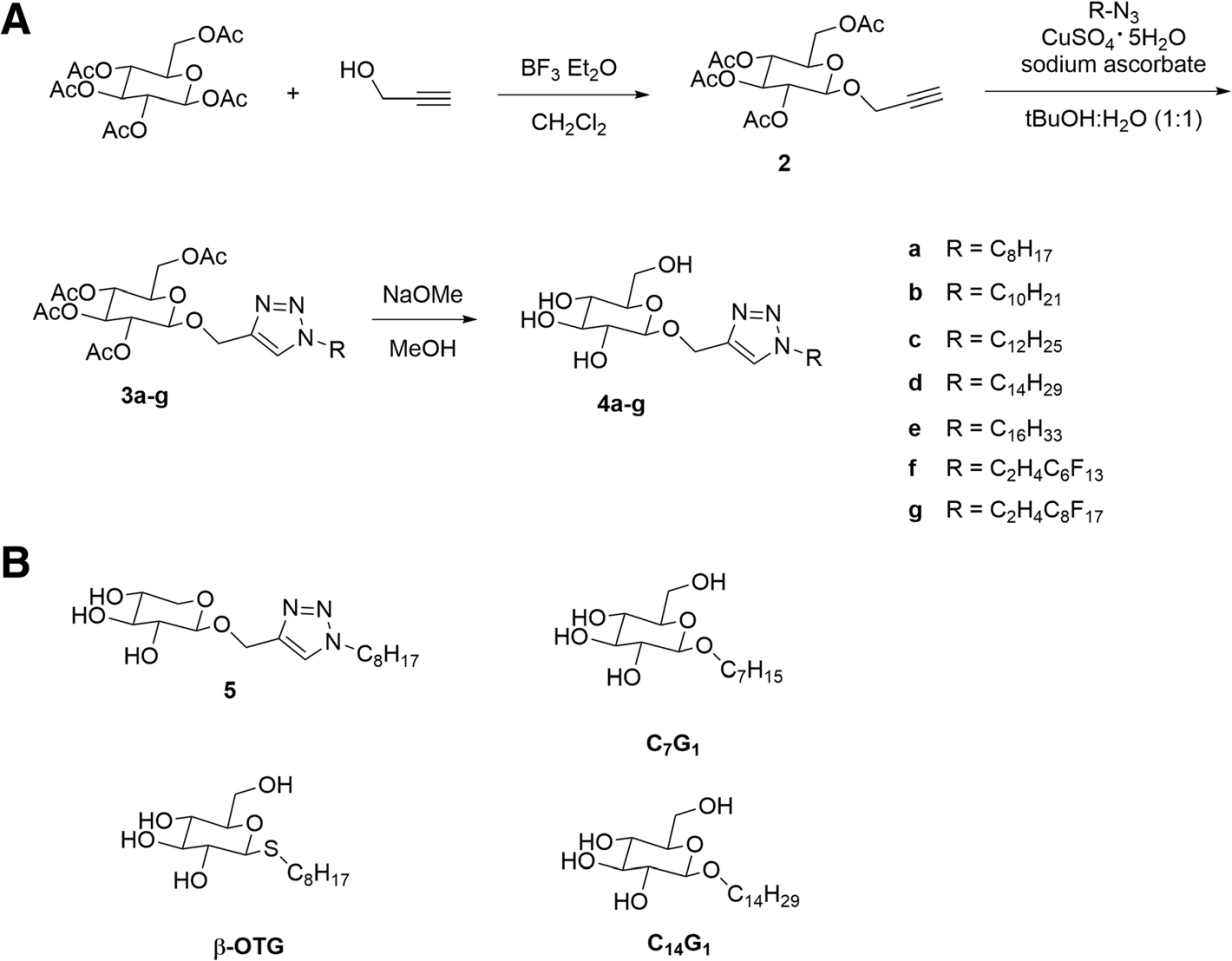
**(A) Synthesis of triazole-containing alkyl β-D-glucopyranosides using the CuAAC reaction; (B) Chemical structure and abbreviations of reference surfactants used in the cell culture studies. 5**, (1-octyl-1*H*-1,2,3-triazol-4-yl)methyl β-D-xylopyranoside; C_7_G_1_, heptyl-β-D-glucopyranoside; β-OTG, 1-*S*-octyl-β-D-thioglucopyranoside; C_14_G_1_, tetradecyl-β-D-glucopyranoside.

The synthesis of the desired glucose-based surfactants was analogous to our previously published synthesis of triazole-containing alkyl β-D-xylopyranosides [9]. These alkyl β-D-xylopyranosides contained a triazole ring incorporated through the CuAAC reaction [8], and possessed surface-active properties. This approach utilizes the ability of this so-called “click” reaction to quickly prepare a series of related molecules. Briefly, the synthesis began with a β-selective glycosylation of commercially-available β-D-glucose pentaacetate (Scheme 1A). This strategy was chosen to yield anomerically pure surfactants, as previous work has suggested the β-alkyl anomers may be more biocompatible [29]. Glycosylation with propargyl alcohol under Lewis-acid-promoted conditions afforded **2** as the β-anomer [30]. The CuAAC reaction between **2** and alkyl azides, which can easily be prepared from the corresponding alkyl bromides or iodides [31], was carried out using 0.1 equiv CuSO_4_ and 0.2 equiv sodium ascorbate in aqueous *tert*-butanol to generate **3a–g** [8]. In the last step the acetate protecting groups were removed with sodium methoxide, followed by neutralization with Dowex 50 W × 8–100 ion exchange resin to yield the triazole-linked surfactants **4a-g**. The final products were purified by recrystallization and provided satisfactory elemental analysis. ^1^H NMR spectroscopy confirmed the anomeric stereochemistry; the only previously reported synthesis of **4a-e** used a Fisher glycosylation which resulted in a mixture of α and β anomers [10]. Overall, the synthetic approach outlined in Scheme 1 offers a facile approach to a large range of novel carbohydrate surfactants with well-defined stereochemistry at the anomeric carbon.

### In vitro cytotoxicity of triazole-containing alkyl xyloside surfactants

Biocompatibility studies using mammalian cells in culture suggests that many carbohydrate surfactants with hydrocarbon and partially fluorinated hydrophobic tails are relatively non-toxic *in vitro*, with no cytotoxicity observable even at millimolar concentrations for some surfactants [4-7,9,13,16,23-29]. Growing experimental evidence suggests that many of these surfactants cause cell death by a mechanism involving apoptosis, not necrosis [9,23,25]. Here, we initially investigated the cytotoxicity of a series of seven triazole-containing alkyl glucopyranosides surfactants **4a-g** (Scheme 1) with the MTS ([3-(4,5-dimethylthiazol-2-yl)-5-(3-carboxymethoxyphenyl)-2-(4-sulfophenyl)-2H-tetrazolium]) assay in the Jurkat cell line and, subsequently, explored possible mechanisms by which they cause cytotoxicity. Four structurally related carbohydrate surfactants (Scheme 1B) were also included in our initial cytotoxicity screening to facilitate the comparison with earlier studies [9,23-25].

The IC_50_ values of the triazole-containing alkyl β-D-glucopyranoside surfactants **4a-e** decreased with increasing alkyl chain length (Table 1), i.e., their cytotoxicity increased with increasing chain-length. Similarly, the cytotoxicity of structurally related, hydrocarbon based carbohydrate surfactants, such as triazole-containing alkyl β-D-xylopyranosides, alkyl β-D-xylopyranosides, alkyl α- and β-D-glactopyranosides, alkyl α- and β-D-glucopyranoside surfactants, and 6-triazole-linked galacto- or glucolipids, increases from short to medium hydrophobic tails [9,16,23-25,29]. One likely explanation for this effect is an increased partitioning of the carbohydrate surfactants into the cell with increasing length of the hydrophobic tail. As a result, the intracellular concentration of homologous carbohydrate surfactants and, thus, their cytotoxicity increases as a function of hydrophobic tail length. Consistent with this observation, we have shown that the apparent membrane partitioning coefficient of carbohydrate surfactants is proportional to the hydrophobic tail length [24]. Unlike hydrocarbon surfactants **4a-e**, many other carbohydrate surfactants investigated to date display a “cut-off” effect [32], i.e., carbohydrate surfactants with a long hydrophobic tail show a decrease in the cytotoxicity relative to medium length surfactant [9,23-25,29]. For example, triazole-containing alkyl β-D-xylopyranosides displayed large IC_50_ values for short chain (hexyl) and long chain surfactants (tetradecyl and hexadecyl), whereas the medium chain alkyl β-D-xylopyranosides (decyl and dodecyl) were the most toxic compounds in this series of surfactants [9]. Although we did not investigate longer hydrophobic tails due to the poor aqueous solubility of the corresponding surfactant (i.e., > hexadecyl), we propose that triazole-containing alkyl β-D-glucopyranoside surfactants **4** would display a “cut-off” effect for surfactants with long hydrophobic tails. The structure-dependent factors likely involved the “cut-off” effect, such as lipid and water solubility, critical aggregate concentration, binding to proteins in the cell and cell culture medium, and diffusion through the cell membrane, are poorly understood and warrant further investigation [32,33].

**Table 1:** **IC**_**50**_ **values of hydrocarbon and fluorocarbon triazole-linked alkyl β-D-glucopyranosides 4a-g tested on Jurkat cells** ^**a**^

**Compound**	**Alkyl β-D-glucopyranosides** ^**b**^							**Control surfactants** ^**b**^			
	**4a**	**4b**	**4c**	**4d**	**4e**	**4f**	**4g**	**5**	**C14G1**	**β-OTG**	**C7G1**
**Hydrophobic tail**	**C**_**8**_ **H**_**17**_	**C**_**10**_ **H**_**21**_	**C**_**12**_ **H**_**25**_	**C**_**14**_ **H**_**29**_	**C**_**16**_ **H**_**33**_	**C**_**2**_ **H**_**4**_ **C**_**6**_ **F**_**13**_	**C**_**2**_ **H**_**4**_ **C**_**8**_ **F**_**17**_	**C**_**8**_ **H**_**17**_	**C**_**14**_ **H**_**29**_	**C**_**8**_ **H**_**17**_	**C**_**7**_ **H**_**15**_
**IC**_**50**_ **value [μM]**	1198	171	89	53	24	*	*	663	67	163	*

^a^The inhibitory concentration 50% (IC_50_) in μM is defined as the concentration of experimental compound required to inhibited 50% of the conversion of MTS to formazan, as compared with the absorbance produced by untreated cells after 16 h of incubation.

^b^Please see Scheme 1 for the chemical structures and corresponding abbreviations for the alkyl β-D-glucopyranosides and the control surfactants.

*Cytotoxicity was <50% at the highest compound concentration tested (2000 μM) and therefore their IC_50_ values could not be determined.

Comparison of the IC_50_ values of the triazole-containing alkyl β-D-glucopyranosides surfactants **4** with structurally related surfactants reveals interesting structure-toxicity relationships (Table 1). For example, the IC_50_ values of the alkyl β-D-glucopyranosides C14G1 is comparable to the IC_50_ values of the structurally related triazole-containing alkyl glucoside **4d**. This observations is consistent with our earlier findings that the triazole-linker does not markedly affect the cytotoxicity of triazole-containing alkyl xyloside [9] and the more general expectation that the introduction of a carbohydrate group renders drug molecules containing a triazole group less cytotoxic [34]. In contrast, the triazole-containing octyl glucoside compound **4a** appeared to be more toxic compared to its structural analogue, C7G1, with IC_50_ values of 1198 μM and > 2,000 μM, respectively. It is also interesting to note that the IC_50_ value of the triazole-containing octyl xyloside **5** was significantly lower compared to its structural glucoside analog **4a**, indicating that xyloside-based surfactants are more cytotoxic compared to glucose-based surfactants. This observation is remarkable because xylose and glucose differ only by a single hydroxymethyl group, but otherwise have the same stereochemistry in the pyranose ring system.

Consistent with this observation, we have previously reported that small changes in the structure of the carbohydrate head group of a surfactant can influence its toxicity [24]. Simple hexadecyl and octadecyl glucopyranoside surfactants, but not structurally related galactoside surfactants caused cytotoxicity at low millimolar concentrations in the B16F10 mouse melanoma cell line. A similar observation has been reported for partially fluorinated gluco- vs. galactopyranoside in the B16 melanoma cell line [28] and for 6-triazole-linked galacto- or glucolipids in the A549 human lung adenocarcinoma epithelial cell line [16]. Moreover, there is some evidence that the configuration at the anomeric center may play a role in the cytotoxicity of carbohydrate surfactants in different cancer cell lines [29]; however this effect of the stereochemistry on the anomeric center has not been observed in all studies [24], possibly due to differences in the carbohydrate surfactants, cell lines and/or experimental conditions employed. Since the stereochemistry of hydroxyl groups of some surfactants, such as uronic acid-based surfactants, is known to drastically alter their physicochemical properties [35,36], it is possible that small changes in the stereochemistry of the polar head-group result in differences in the cytotoxicity by either indirectly altering physicochemical properties of macromolecular structures, such as the cell membrane, and/or direct interaction with cellular targets.

The two partially fluorinated surfactants **4f** (F-octyl) and **4g** (F-decyl) displayed no cytotoxicity in the Jurkat cell line over the entire concentration range investigated (8 μM to 2 mM). The hydrocarbon surfactant **4a**, which is the structural analog of partially fluorinated surfactant **4g**, displayed low toxicity in the Jurkat cell line, with an IC_50_ value of 1,198 μM. Similarly, many other studies have reported that the introduction of a perfluoroalkyl group in a hydrocarbon surfactant is typically protective and significantly decreases its cytotoxicity in mammalian cells in culture [5-7,9,24,25,27,28]. However, a perfluoroalkyl group in the hydrophobic tail is not always protective, as we have shown for octyl versus F-octyl β-D-xylopyranosides [23]. These differences in the cytotoxicity of hydrocarbon and partially fluorinated carbohydrate surfactants likely reflect differences in the physicochemical properties of the respective carbohydrate surfactant caused by the introduction of varying degrees of fluorination in the hydrophobic tail.

### Annexin V/PI apoptosis/necrosis assay

**Figure 1:**
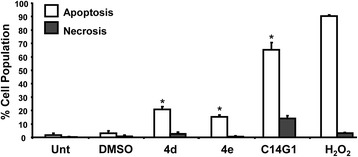
**Triazole-containing alkyl β-D-glucopyranosides 4d and 4e and alkyl β-D-glucopyranosides C14G1 induced significant phosphatidylserine externalization in the Jurkat cell line.** The mode of cell death induction, apoptosis or necrosis, was monitored *via* flow cytometric assay after co-staining of cells with Annexin V-FITC and PI. Cells were exposed to the ~ IC_50_ concentration of each compound as determined by MTS assay (see Table 1). The total percentage of apoptotic cells is expressed as the sum of percentages of both early and late stages of apoptosis (Annexin V-FITC positive; white bars), with green fluorescence signal. Cells that were stained only with PI due to the loss of plasma membrane integrity, but without FITC signal, are considered necrotic cells (gray bars). Analysis of the apoptotic populations using the two-tailed Student's paired *t*-test of **4d**, **4e** and C14G1-treated Jurkat cells against DMSO and untreated controls was *P* < 0.001 (*). Each bar represents the average of three independent measurement values, and error bars represent the standard deviation of the mean. Unt refers to untreated cells.

Our previous studies demonstrate that structurally diverse carbohydrate surfactants, including triazole-linked alkyl β-D-xylopyranosides cause cytotoxicity by apoptosis and not necrosis [9,23,25]. We therefore assessed whether triazole-linked alkyl β-D-glucopyranosides **4d** (tetradecyl) and **4e** (hexadecyl) cause cytotoxicity by apoptosis or necrosis. Because phosphatidylserine translocation from the inner leaflet to the outer membrane is an early event in apoptotic cell death [37], Annexin V-FITC, which has a high affinity for phosphatidylserine, was used to detect phosphatidylserine as a marker of apoptosis in live-cells by flow cytometry. As can be seen in Figure 1, a significant amount of phosphatidylserine is externalized when Jurkat cells were treated with the tetradecyl glycopyranoside C14G1 (~65%), while treatment with **4d** and **4e** resulted in lower (<20%) but significant Annexin-V staining after a 16 h incubation. These findings demonstrate that, similar to other carbohydrate surfactants, triazole-linked alkyl glucopyranosides **4d** and **4e** cause apoptosis in the Jurkat cell line.

### Surfactant-mediated cytotoxicity involves a mitochondria-dependent apoptosis pathway

**Figure 2:**
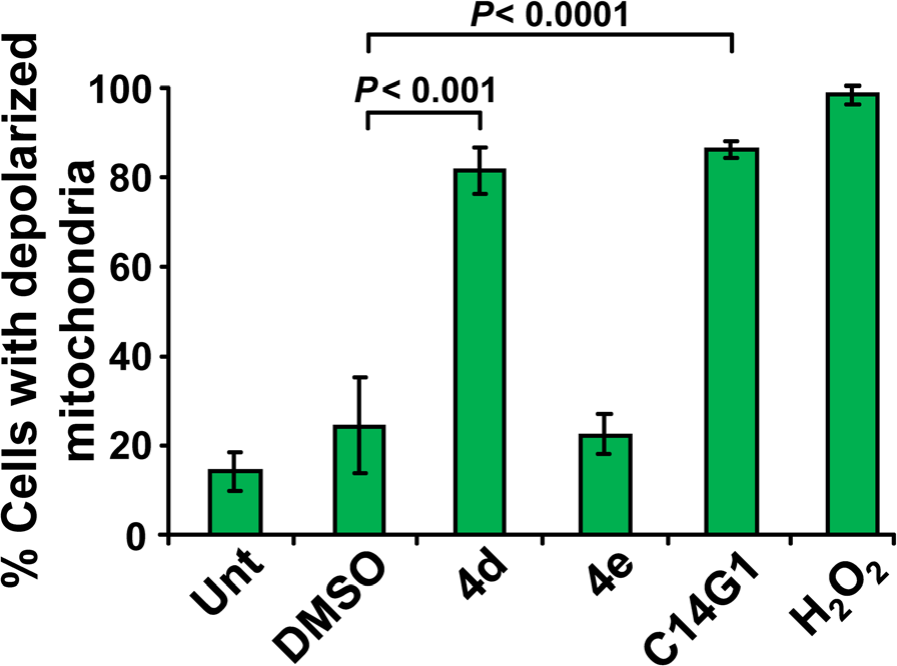
**Mitochondrial depolarization mediated by triazole-containing alkyl β-D-glucopyranosides 4d and 4e and alkyl β-D-glucopyranosides C14G1.** Jurkat cells were treated with triazole-containing alkyl β-D-glucopyranosides **4d** and **4e** and alkyl β-D-glucopyranosides C14G1 at their respective IC_50_ concentration and incubated for 6 h. Changes in the mitochondrial *ΔΨm* was determined by staining with 2 μM of JC-1. The IC_50_ concentration values that were used were the following: **4d** = 53 μM; **4e** = 24 μM; and C14G1 = 66.5 μM. After dissipation of *ΔΨm*, the JC-1 reagent emits a green fluorescence signal, while the compound in a polarizedmitochondrial membrane emits a red signal. Percentages of cells emitting green fluorescence signal (*y*-axis) are depicted. Each bar represents the mean ± SD of four independent replicates. The following controls were included: untreated cells as a negative control; cells treated with 0.1% v/v DMSO as a control for solvent effects; and cells exposed to 1 mM H_2_O_2_ as a positive control. Approximately 1x10^4^ flow cytometry events were acquired and analyzed per sample using CXP software.

Apoptosis can be caused by the activation of cysteine-aspartic acid proteases (caspases) through an intrinsic, mitochondria-mediated pathway or an extrinsic pathway involving cellular death receptors, such as FAS/CD95 or tumor necrosis factor receptor 1 (TNFR1). To gain further insights into the mechanisms involved in carbohydrate surfactant-mediated apoptosis, we investigated the dissipation of mitochondrial membrane potential (ΔΨm), an early facet in apoptosis that has been implicated in initiating the intrinsic pathway [38]. Briefly, Jurkat cells were treated for 6 h with carbohydrate surfactants **4d**, **4e** or C14G1, stained with the fluorophore 5,5',6,6'-tetrachloro-1,1',3,3'-tetraethylbenzimidazolylcarbocyanine iodide (JC-1) and analyzed via flow cytometry [39,40]. Results indicated that **4d** and C14G1 compounds provoked preferential accumulation of JC-1 monomers (green), an indicator of mitochondrial depolarization, and interfered with the formation of JC-1 aggregates (red) (Figure 2). A similar distribution pattern was observed in cells treated with the positive control, H_2_O_2_. The most potent compound causing mitochondrial depolarization was C14G1 followed by **4d**. These outcomes suggest that **4d**- and C14G1-mediated cytotoxicity is initiated *via ΔΨm* depolarization, involving the intrinsic apoptotic pathway in the initiation of cell death. In contrast, **4e**-mediated toxicity appeared to circumvent *ΔΨm* dissipation to induce cell death.

### Surfactants inflict cytotoxicity via caspase-3 activation

**Figure 3:**
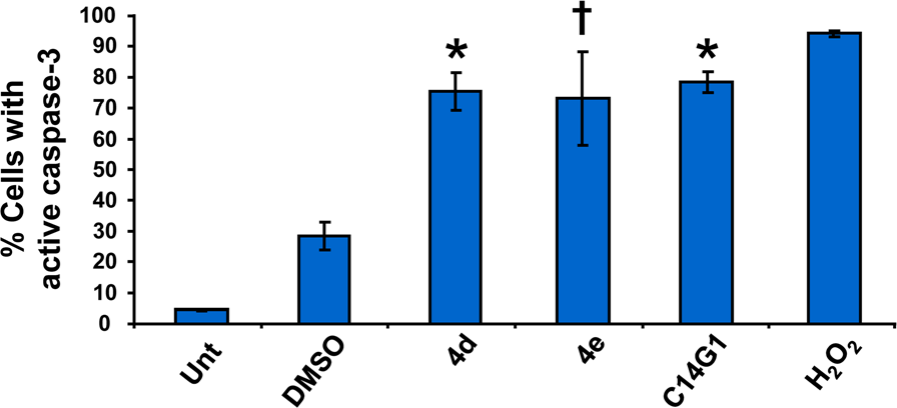
**Treatment of Jurkat cells with triazole-containing alkyl β-D-glucopyranosides 4d and 4e and alkyl β-D-glucopyranosides C14G1 resulted in caspase-3 activation.** Jurkat cells were treated with compounds at their respective IC_50_ concentration. The percentage of cells with activated caspase-3 as determined by emission of green fluorescence signal is indicated on *y* axis. Each bar represents average of three independent measurement values, and error bars their corresponding SD values. Data were analyzed using the two-tailed Student's paired *t*-test of compound treated cells vs. DMSO treated cells with *P*-values within statistically significant range of *P* < 0.0001 (*) and *P* < 0.008 (†).

Caspase-3 is activated by both the intrinsic and extrinsic apoptotic pathways. To examine whether caspase-3 activation was involved in the cytotoxicity provoked by the selected experimental compounds, a cell permeable fluorogenic reagent, NucView 488 Caspase-3 substrate, and Jurkat cells were utilized. This substrate allows the detection of caspase-3 activity in live cells *via* flow cytometry. Jurkat cells with active caspase-3 were significantly detected after 6 h of incubation with **4d**, **4e** and alkyl β-D-glucopyranoside C14G1, as compared with untreated and solvent controls (DMSO; *P* < 0.001; Figure 3) [39,40]. The most efficient carbohydrate surfactant eliciting caspase-3 activation was C14G1 (Figure 3). These observations suggest that the cytotoxicity induced by **4d**, **4e** and C14G1was indeed mediated via apoptosis as initially detected by phosphatidylserine externalization and corroborated by caspase-3 activation; both hallmarks of apoptosis.

## Experimental

### General procedures

The ^1^H and ^13^C NMR spectra were recorded on a Bruker DRX 400 Digital NMR spectrometer. ^19^F spectra were recorded using a Bruker Avance 300. NMR assignments were determined from a COSY spectrum of **3b**. Representative ^1^H and ^13^C NMR spectra are included in Additional file 1. High resolution mass spectra were obtained at the University of California, Riverside Mass Spectrometry facility. Elemental analyses were obtained from Atlantic Micro Lab Microanalysis Service (Atlanta, Georgia, USA). All reactions were monitored by thin layer chromatography, followed by visualization with UV and anisaldehyde-H_2_SO_4_. Azides were prepared using a known method [31] and used without further purification. β-Propargyl 2,3,4,6-tetra-*O*-acetylglucopyranoisde was prepared from commercially available β-D-glucopyranoside pentaacetate as previously described. Compounds **3a-e** and **4a-e** have been reported in the literature [10]. Tetradecyl β-D-glucopyranoside (C14G1) was prepared as previously described [24]. 1-*S*-Octyl-β-D-thioglucopyranoside (β-OTG), propargyl alcohol and Dowex® 50W × 8-100 ion exchange resin were obtained from Acros Organics/Fisher Scientific (Pittsburgh, PA). Boron trifluoride diethyl ethereate and sodium methoxide were obtained from Alfa Aesar (Ward Hill, MA). Sodium azide and sodium ascorbate were obtained from Aldrich (St. Louis, MO). Cupric sulfate pentahydrate was obtained from Mallinckrodt (St. Louis, MO). All organic solvents were reagent grade or higher and were used without further purification. Flash chromatography was performed using 60 Å (40-63 μm, 230x400 mesh) silica gel.

### General procedure for the CuAAC reaction

Triacetyl propargyl glucose (**2**) and n-alkyl azide (1.0 – 1.1 eq.) were combined with 2:1 *tert*-butanol: water (0.33 M) at room temperature. Sodium ascorbate (0.2 eq., 1.0 M in water) was added, followed by CuSO_4_ pentahydrate (0.1 eq., 75 mg/mL in water), and the mixture stirred at room temperature for 90 minutes. At this time the reactions often became homogeneous and faint blue. The reaction mixture was diluted with water and extracted three times with ethyl acetate. The combined extracts were washed with brine, dried over MgSO_4_ and concentrated. The crude residue was purified by silica gel column chromatography (hexanes/EtOAc), or used without further purification.

### (1-Octyl-1*H*-1,2,3-triazol-4-yl)methyl 2,3,4-tri-*O*-acetyl-β-glucopyranoside (3a)

The general procedure was used with propargyl glucose (512 mg, 1.32 mmol) and 1-azidooctane (208 mg, 1.32 mmol); after column chromatography using EtOAc:hexanes (2:1, v/v), 636 mg (89%) of **3a** were obtained as a clear oil which solidified upon standing. ^1^H NMR (CDCl_3_, 400MHz): δ 7.49 (s, 1H, triazole-CH), 5.18 (app t, *J* = 9.4 Hz, 1H, H-3), 5.08 (app t, *J* = 9.9 Hz, 1H, H-4), 4.99 (dd, *J* = 9.5, 8.0 Hz, 1H, H-2), 4.92 (d, *J* = 12.6 Hz, 1H, H-1’a), 4.80 (d, *J* = 12.6 Hz, 1H, H-1’b), 4.66 (d, *J* = 7.9 Hz, 1H, H-1), 4.32 (t, *J* = 7.3 Hz, 2H, H-α), 4.26 (dd, *J* = 12.3, 4.8 Hz, 1H, H-6a), 4.14 (dd, *J* = 12.3, 2.3 Hz, 1H, H-6b), 3.72 (ddd, *J* = 9.9, 4.6, 2.3 Hz, 1H, H-5), 2.07 (s, 3H, OAc), 2.00 (s, 3H, OAc), 1.98 (s, 3H, OAc), 1.96 (s, 3H, OAc), 1.84 - 1.91 (m, 2H, H-β), 1.15 - 1.37 (m, 10H, 5 × CH_2_), 0.85 (t, *J* = 6.5 Hz, 3H, H-ω); ^13^C NMR (CDCl_3_, 100 MHz): δ 170.6, 170.2, 169.4, 169.3, 144.0, 122.5, 99.7, 72.7, 71.8, 71.1, 68.1, 63.0, 61.7, 50.4, 31.6, 30.2, 29.0, 28.8, 26.4, 22.5, 20.7, 20.61, 20.56 (2 × C), 14.0; HRESIMS calcd for C_25_H_40_N_3_O_10_ (M + H)^+^: 542.2708; found: 542.2710.

### (1-Decyl-1*H*-1,2,3-triazol-4-yl)methyl 2,3,4-tri-*O*-acetyl-β-glucopyranoside (3b)

The general procedure was used with propargyl glucose (507 mg, 1.31 mmol) and 1-azidodecane (240 mg, 1.31 mmol); after column chromatography using EtOAc:hexanes (2:1, v/v), 669 mg (90%) of **3b** were obtained as a waxy solid. ^1^H NMR (CDCl_3_, 400MHz): δ 7.50 (s, 1H, triazole-CH), 5.19 (app t, *J* = 9.4 Hz, 1H, H-3), 5.09 (app t, *J* = 9.8 Hz, 1H, H-4), 5.01 (dd, *J* = 9.5, 8.0 Hz, 1H, H-2), 4.93 (d, *J* = 12.5 Hz, 1H, H-1’a), 4.82 (d, *J* = 12.5 Hz, 1H, H-1’b), 4.68 (d, *J* = 8.0 Hz, 1H, H-1), 4.33 (t, *J* = 7.3 Hz, 2H, H-α), 4.27 (dd, *J* = 12.3, 4.8 Hz, 1H, H-6a), 4.15 (dd, *J* = 12.3, 2.3 Hz, 1H, H-6b), 3.73 (ddd, *J* = 9.9, 4.7, 2.3 Hz, 1H, H-5), 2.09 (s, 3H, OAc), 2.02 (s, 3H, OAc), 1.99 (s, 3H, OAc), 1.98 (s, 2H, OAc), 1.84 - 1.94 (m, 2H, H-β), 1.18 - 1.37 (m, 14H, 7 × CH_2_), 0.87 (t, *J* = 6.5 Hz, 3H, H-ω); ^13^C NMR (CDCl_3_, 100 MHz): δ 170.5, 170.0, 169.3, 169.2, 143.3, 122.4, 99.5, 72.7, 71.8, 71.1, 68.2, 62.9, 61.7, 50.3, 31.7, 30.2, 29.35, 29.25, 29.1, 28.9, 26.4, 22.5, 20.6, 20.53, 20.47 (2 × C), 14.8; HRESIMS calcd for C_27_H_44_N_3_O_10_: 570.3021; found: 570.3034.

### (1-Dodecyl-1*H*-1,2,3-triazol-4-yl)methyl 2,3,4-tri-*O*-acetyl-β-glucopyranoside (3c)

The general procedure was used with propargyl glucose (526 mg, 1.36 mmol) and 1-azidododecane (286 mg, 1.36 mmol); after column chromatography using EtOAc:hexanes (2:1, v/v), 666 mg (82%) of **3c** were obtained as a waxy solid. ^1^H NMR (CDCl_3_, 400MHz): δ 7.50 (s, 1H, triazole-CH), 5.20 (app t, *J* = 9.5 Hz, 1H, H-3), 5.09 (app t, *J* = 9.9 Hz, 1H, H-4), 5.01 (dd, *J* = 9.5, 7.9 Hz, 1H, H-2), 4.94 (d, *J* = 12.5 Hz, 1H, H-1’a), 4.82 (d, *J* = 12.5 Hz, 1H, H-1’b), 4.69 (d, *J* = 8.0 Hz, 1H, H-1), 4.33 (t, *J* = 7.2 Hz, 2H, H-α), 4.27 (dd, *J* = 12.3, 4.8 Hz, 1H, H-6a), 4.15 (dd, *J* = 12.3, 2.4 Hz, 1H, H-6b), 3.73 (ddd, *J* = 10.0, 4.8, 2.4 Hz, 1H, H-5), 2.09 (s, 3H, OAc), 2.03 (s, 3H, OAc), 2.00 (s, 3H, OAc), 1.98 (s, 3H, OAc), 1.85-1.95 (m, 2H, H-β), 1.19 - 1.36 (m, 18H, 9 x CH_2_), 0.88 (t, *J* = 6.7 Hz, 3H, H-ω); ^13^C NMR (CDCl_3_, 100 MHz) δ 171.7, 171.2, 170.5, 170.4, 145.1, 123.5, 101.0, 73.9, 73.0, 72.3, 69.4, 64.1, 62.9, 51.5, 32.9, 31.4, 30.63 (2 × C), 30.56, 30.43, 30.36, 30.0, 27.6, 23.7, 21.8, 21.7, 21.6 (2 × C), 15.2; HRESIMS calcd for C_29_H_48_N_3_O_10_: 598.3334; found: 598.3339.

### (1-Tetradecyl-1*H*-1,2,3-triazol-4-yl)methyl 2,3,4-tri-*O*-acetyl-β-glucopyranoside (3d)

The general procedure was used with propargyl glucose (526 mg, 1.36 mmol) and 1-azidotetradecane (371 mg, 1.55 mmol); after column chromatography using EtOAc:hexanes (3:2, v/v), 636 mg (75%) of **3d** were obtained as a waxy solid. ^1^H NMR (CDCl_3_, 400MHz): δ 7.49 (s, 1H, triazole-CH), 5.19 (app t, *J* = 9.4 Hz, 1H, H-3), 5.09 (app t, *J* = 9.9 Hz, 1H, H-4), 5.01 (dd, *J* = 9.5, 8.0 Hz, 1H, H-2), 4.93 (d, *J* = 12.5 Hz, 1H, H-1’a), 4.81 (d, *J* = 12.1 Hz, 1H, H-1’b), 4.68 (d, *J* = 8.1 Hz, 1H, H-1), 4.33 (t, *J* = 7.3 Hz, 2H, H-α), 4.27 (dd, *J* = 12.3, 4.8 Hz, 1H, H-6a), 4.14 (dd, *J* = 12.3, 2.3 Hz, 1H, H-6b), 3.69 - 3.78 (m, 1H, H-5), 2.08 (s, 3H, OAc), 2.02 (s, 3H, OAc), 1.99 (s, 3H, OAc), 1.97 (s, 3H, OAc), 1.83 - 1.94 (m, 2H, H-β), 1.17 - 1.40 (m, 22H, 11 x CH_2_), 0.87 (t, *J* = 6.8 Hz, 3H, H-ω); ^13^C NMR (CDCl_3_, 100 MHz): δ 170.6, 170.1, 169.4, 169.3, 144.0, 122.4, 99.8, 72.7, 71.9, 71.2, 68.3, 63.0, 61.8, 50.4, 31.9, 30.3, 29.6, 29.6, 29.58 (2 × C), 29.54, 29.33, 29.29, 28.9, 26.5, 22.6, 20.7, 20.6, 20.5 (2 × C), 14.1; HRESIMS calcd for C_31_H_52_N_3_O_10_: 626.3647; found: 626.3670.

### (1-Hexadecyl-1*H*-1,2,3-triazol-4-yl)methyl 2,3,4-tri-*O*-acetyl-β-glucopyranoside (3e)

The general procedure was used with propargyl glucose (528 mg, 1.36 mmol) and 1-azidohexadecane (373 mg, 1.38 mmol); after column chromatography using EtOAc:hexanes (3:2, v/v), 692 mg (78%) of **3e** were obtained as a white solid. ^1^H NMR (CDCl_3_, 400MHz): δ 7.49 (s, 1H, triazole-CH), 5.19 (app t, *J* = 9.5 Hz, 1H, H-3), 5.08 (app t, *J* = 9.8 Hz, 1H, H-4), 5.00 (dd, *J* = 9.5, 8.0 Hz, 1H, H-2), 4.93 (d, *J* = 12.6 Hz, 1H, H-1’a), 4.81 (d, *J* = 12.5 Hz, 1H, H-1’b), 4.68 (d, *J* = 7.9 Hz, 1H, H-1), 4.32 (t, *J* = 7.2 Hz, 2H, H-α), 4.27 (dd, *J* = 12.3, 4.8 Hz, 1H, H-6a), 4.14 (dd, *J* = 12.3, 2.3 Hz, 1H, H-6b), 3.73 (ddd, *J* = 9.9, 4.7, 2.4 Hz, 1H, H-5), 2.08 (s, 3H, OAc), 2.01 (s, 3H, OAc), 1.98 (s, 3H, OAc), 1.97 (s, 3H, OAc), 1.81 - 1.94 (m, 2H, H-β), 1.16 - 1.36 (m, 26H, 13 × CH_2_), 0.87 (t, *J* = 6.6 Hz, 3H, H-ω); ^13^C NMR (CDCl_3_, 100 MHz): δ 170.6, 170.1, 169.4, 169.3, 143.9, 122.5, 99.7, 72.7, 71.8, 71.1, 68.2, 62.9, 61.7, 50.3, 31.8, 30.9, 30.2, 29.59 (2 × C), 29.56 (2 × C), 29.52, 29.45, 29.3, 29.3, 28.9, 26.4, 22.6, 20.7, 20.6, 20.5, 14.1; HRESIMS calcd for C_33_H_56_N_3_O_10_: 654.3960; found: 654.3980.

### (1-(3,3,4,4,5,5,6,6,7,7,8,8,8-Tridecafluorooctyl)-1*H*-1,2,3-triazol-4-yl)methyl 2,3,4-tri-*O*-acetyl-β-glucopyranoside (3f)

The general procedure was used with propargyl glucose (702 mg, 1.81 mmol) and 1-azido-3,3,4,4,5,5,6,6,7,7,8,8,8-tridecafluorooctane (707 mg, 1.81 mmol); after column chromatography using EtOAc:hexanes (3:2, v/v), 846 mg (60%) of **3f** were obtained as a white solid. ^1^H NMR ^1^H NMR (CDCl_3_, 400 MHz): δ 7.60 (s, 1H, triazole-CH), 5.20 (app t, *J =* 9.3 Hz, 1H, H-3), 5.10 (app t, *J =* 9.8 Hz, 1H, H-4), 5.01 (dd, *J* = 9.4, 8.1 Hz, 1H, H-2), 4.94 (d, *J* = 12.4 Hz, 1H, H-1’a), 4.83 (d, *J* = 12.7 Hz, 1H, H-1’b), 4.66-4.69 (m, 3H, H-1, H-α), 4.25 (dd, *J* = 12.4, 4.6 Hz, 1H, H-6a), 4.15 (dd, *J* = 12.4, 2.1 Hz, 1H, H-6b), 3.73 (ddd, *J* = 10.0, 4.4, 2.3 Hz, 1H, H-5), 2.83 (tt, *J* = 18.0, 7.5 Hz, 1H, H-β); ^13^C NMR (CDCl_3_, 100 MHz): δ 170.6, 170.2, 169.4, 169.4, 144.6, 123.4, 100.0, 72.6, 71.9, 71.1, 68.2, 63.0, 61.6, 42.3, 31.6, 20.7, 20.6 (3 × C); ^19^F NMR (282 MHz, CDCl_3_) δ -81.23, -114.70, -122.35, -123.37, -123.96, -126.64; HRESIMS calcd for C_25_H_27_N_3_O_10_F_13_: 776.1483; found: 776.1470.

### (1-(3,3,4,4,5,5,6,6,7,7,8,8,9,9,10,10,10-Heptadecafluorodecyl)-1*H*-1,2,3-triazol-4-yl)methyl 2,3,4-tri-*O*-acetyl-β-glucopyranoside (3g)

The general procedure was used with propargyl glucose (702 mg, 1.81 mmol) and 1-azido-3,3,4,4,5,5,6,6,7,7,8,8,9,9,10,10,10-heptadecafluorooctane (707 mg, 1.81 mmol); after column chromatography using EtOAc:hexanes (3:2, v/v), 846 mg (60%) of **3g** were obtained as a white solid. ^1^H NMR (CDCl_3_, 400 MHz): δ 7.59 (s, 1H, triazole-CH), 5.20 (app t, *J =* 9.3 Hz, 1H, H-3), 5.09 (app t, *J =* 9.8 Hz, 1H, H-4), 5.01 (dd, *J* = 9.4, 8.1 Hz, 1H, H-2), 4.93 (d, *J* = 12.4 Hz, 1H, H-1’a), 4.83 (d, *J* = 12.7 Hz, 1H, H-1’b), 4.66-4.70 (m, 3H, H-1, H-α), 4.25 (dd, *J* = 12.4, 4.6 Hz, 1H, H-6a), 4.18 (dd, *J* = 12.4, 2.1 Hz, 1H, H-6b), 3.73 (ddd, *J* = 10.0, 4.4, 2.3 Hz, 1H, H-5), 2.83 (tt, *J* = 18.0, 7.5 Hz, 1H, H-β); ^13^C NMR (CDCl_3_, 100 MHz): δ 170.6, 170.2, 169.4, 169.4, 144.6, 123.4, 100.0, 72.6, 71.9, 71.1, 68.2, 63.0, 61.6, 42.3, 31.6, 20.7, 20.6 (3 × C); ^19^F NMR (282 MHz, CDCl_3_) δ -81.23, -114.68, -122.13, -122.44 (2 × CF_2_), -123.22, -123.94, -126.62; HRESIMS calcd for C_27_H_27_N_3_O_10_F_17_: 867.1420; found: 876.1421.

### General procedure for acetate deprotection

Triazole peracetates **3** were stirred in dry methanol. NaOMe (1 eq.) was added and the solution stirred at room temperature for 2-4 hr. Dowex® 50W × 8-100 ion exchange resin was added and the reaction mixture stirred for another 30 min. The resin was filtered and the solvent concentrated. The crude residue was purified by recrystallization or column chromatography to yield pure 1-alkyl-1*H*-1,2,3-triazol-4-ylmethyl β-D-glucopyranosides **4**.

### (1-Octyl-1*H*-1,2,3-triazol-4-yl)methyl β-D-glucopyranoside (4a)

Following the general procedure for acetate deprotection, 902 mg (1.66 mmol) of **3a** and 90 mg (1.66 mmol) NaOMe were stirred in 6 mL MeOH. The crude product purified by column chromatography, yielding 434 mg (70%) of **4a** as a white solid. ^1^H NMR (MeOD, 400 MHz): δ 8.03 (s, 1H, triazole-CH), 4.98 (d, *J* = 12.3 Hz, 1H, H-1’a), 4.79 (d, *J* = 12.4 Hz, 1H, H-1’b), 4.37 - 4.43 (m, 3H, H-1, H-α), 3.91 (dd, *J* = 11.9, 1.7 Hz, 1H, H-6a), 3.69 (dd, *J* = 11.9, 5.6 Hz, 1H, H-6b), 3.18 - 3.41 (m, 4H, H-2, H-3, H-4, H-5), 1.85 - 1.98 (m, 2H, H-β), 1.22 - 1.42 (m, 10H, 5 × CH_2_), 0.90 (t, *J* = 6.7 Hz, 3H, H-ω); ^13^C NMR (MeOD, 100 MHz): δ 145.8, 125.4, 103.7, 78.2, 78.1, 75.1, 71.7, 63.1, 62.9, 51.5, 33.1, 31.5, 30.4, 30.2, 27.6, 23.8, 14.6; HRESIMS calcd for C_17_H_32_N_3_O_6_ (M + H)^+^: 374.2286; found: 374.2289; Anal calcd for C_17_H_31_N_3_O_6_: C 54.68, H 8.37, N 11.25; found: C 54.43, H 8.20, N 10.99.

### (1-Decyl-1*H*-1,2,3-triazol-4-yl)methyl β-D-glucopyranoside (4b)

Following the general procedure for acetate deprotection, 597 mg (1.05 mmol) of **3b** and 56 mg (1.05 mmol) NaOMe were stirred in 4 mL MeOH for 4 hours. The crude product was purified by column chromatography (5:4:1 CH_2_Cl_2_:acetone:MeOH), yielding 266 mg (62%) of **4b** as a white solid. ^1^H NMR (MeOD, 400 MHz): δ 8.03 (s, 1H, triazole-CH), 4.78 (d, *J* = 12.4 Hz, 1H, H-1’a), 4.78 (d, *J* = 12.4 Hz, 1H, H-1’b), 4.36-4.44 (m, 3H, H-1, H-α), 3.90 (dd, *J* = 11.9, 1.6 Hz, 1H, H-6a), 3.68 (dd, *J* = 11.9, 5.6 Hz, 1H, H-6b), 3.11 - 3.44 (m, 4H, H-2, H-3, H-4, H-5), 1.81 - 2.00 (m, 2H, H-β), 1.17 - 1.44 (m, 14H, 7 × CH_2_), 0.90 (t, *J* = 6.7 Hz, 3H, H-ω); ^13^C NMR (MeOD, 100 MHz) δ 145.8, 125.4, 103.7, 78.2, 78.1, 75.1, 71.7, 63.1, 62.9, 51.5, 33.2, 31.4, 30.8, 30.7, 30.6, 30.3, 27.6, 23.9, 14.6; HRESIMS calcd for C_19_H_36_N_3_O_6_ (M + H)^+^: 402.2599; found: 402.2599; Anal cald for C_19_H_35_N_3_O_6_ (H_2_O)_0.4_: C 55.84, H 8.83, N 10.28; found: C 55.90, H 8.77, N 10.09.

### (1-Dodecyl-1*H*-1,2,3-triazol-4-yl)methyl β-D-glucopyranoside (4c)

Following the general procedure for acetate deprotection, 540 mg (0.904 mmol) of **3c** and 48 mg (0.904 mmol) NaOMe were stirred in 4 mL MeOH for 4 hours. The crude product was purified by recrystallization from acetone/hexane, yielding 266 mg (62%) of **4c** as a white solid. ^1^H NMR (MeOD, 400 MHz): δ 8.01 (s, 1H, triazole-CH), 4.97 (d, *J* = 12.3 Hz, 1H, H-1’a), 4.78 (d, *J* = 12.4 Hz, 1H, H-1’b), 4.38-4.1 (m, 3H, H-1, H-α), 3.90 (dd, *J* = 11.7, 1.5 Hz, 1H, H-6a), 3.66 (dd, *J* = 11.9, 4.9 Hz, 1H, H-6b), 3.17 - 3.37 (m, 4H, H-2, H-3, H-4, H-5), 1.78 - 2.04 (m, 2H, H-β), 1.19 - 1.42 (m, 18H, 9 x CH_2_), 0.90 (t, *J* = 6.7 Hz, 3H, H-ω); ^13^C NMR (MeOD, 100 MHz): δ 145.8, 125.4, 103.8, 78.2, 78.1, 75.2, 71.8, 63.2, 62.9, 51.5, 33.2, 31.4, 30.9 (2 × C), 30.8, 30.7, 30.6, 30.2, 27.6, 23.9, 14.6; HRESIMS calcd for C_21_H_40_N_3_O_6_ (M + H)^+^: 430.2912; found: 430.2917; Anal calcd for C_21_H_39_N_3_O_6_(H_2_O)_0.25_: C 58.11, H 9.17, N 9.68: Found: C 58.33, H 9.14, N 9.37.

### (1-Tetradecyl-1*H*-1,2,3-triazol-4-yl)methyl β-D-glucopyranoside (4d)

Following the general procedure for acetate deprotection, 394 mg (0.630 mmol) of **3d** and 34 mg (0.63 mmol) NaOMe were stirred in 2.4 mL MeOH for 2 hours. The crude product was purified by column chromatography (5:4:1 CH_2_Cl_2_:acetone:MeOH), yielding 177 mg (61%) of **4d** as a white solid. ^1^H NMR (MeOD, 400MHz): δ 8.01 (s, 1H, triazole-CH), 4.97 (d, *J* = 12.4 Hz, 1H, H-1’a), 4.78 (d, *J* = 12.4 Hz, 1H, H-1’b), 4.35 - 4.44 (m, 3H, H-1, H-α), 3.90 (dd, *J* = 11.8, 1.6 Hz, 1H, H-6a), 3.65 - 3.72 (m, 1H, H-6b), 3.19 - 3.34 (m, 4H, H-2, H-3, H-4, H-5), 1.83 - 1.99 (m, 2H, H-β), 1.18 - 1.43 (m, 22H, 11 × CH_2_), 0.90 (t, *J* = 6.6 Hz, 3H, H-ω); ^13^C NMR (MeOD, 100 MHz): δ 145.8, 125.4, 103.8, 78.2, 78.1, 75.2, 71.8, 63.2, 62.9, 51.5, 33.2, 31.4, 30.92, 30.90, 30.89, 30.87, 30.8, 30.7, 30.6, 30.2, 27.6, 23.9, 14.6; HRESIMS calcd for C_24_H_44_N_3_O_6_ (M + H)^+^: 458.3225; found: 458.3246; Anal calcd for C_24_H_43_N_3_O_6_: 60.37, H 9.47, N 9.18; found: C 59.97, H 9.29, N 8.91.

### (1-Hexadecyl-1*H*-1,2,3-triazol-4-yl)methyl β-D-glucopyranoside (4e)

Following the general procedure for acetate deprotection, 800 mg (1.22 mmol) of **3e** and 66 mg NaOMe (1.22 mmol) were stirred in 5 mL MeOH for 3.5 hours. The crude product was purified by recrystallization from methanol, yielding 176 mg (30%) of **4e** as a white solid. ^1^H NMR (DMSO-d_6_, 400 MHz): δ 8.10 (s, 1H, triazole-CH), 4.83 (d, *J* = 12.1 Hz, 1H, H-1’a), 4.62 (d, *J* = 12.4 Hz, 1H, H-1’b), 4.32 (t, *J* = 7.1 Hz, 2H, H-α), 4.25 (d, *J* = 7.7 Hz), 3.71 (dd, *J* = 11.7, 1.8 Hz, 1H, H-6a), 3.46 (dd, *J* = 11.8, 6.4 Hz, 1H, H-6b), 3.08 - 3.18 (m, 3H, H-3, H-4, H-5), 2.98 (app t, *J* = 7.8 Hz, H-2), 1.73 - 1 .86 (m, 2H, H-β), 1.15 - 1.39 (m, 26H, 13 × CH_2_), 0.85 (t, *J* = 7.3 Hz, H-ω); ^13^C NMR (DMSO-d_6_, 100 MHz): δ 143.7, 124.0, 102.1, 76.9, 76.7, 73.4, 70.1, 61.5, 61.2, 49.2, 31.3, 29.7, 29.01 (4 × C), 28.98 (2 × C), 28.93, 28.85, 28.7, 28.4, 25.8, 22.0, 13.9; HRESIMS calcd for C_25_H_48_N_3_O_6_ (M + H)^+^: 486.3538; found: 486.3530; Anal calcd for C_25_H_47_N_3_O_6_ : C 61.83, H 9.75, N 8.65; found: C 61.66, H 9.61, N 8.44.

### (1-(3,3,4,4,5,5,6,6,7,7,8,8,8-Tridecafluorooctyl)-1*H*-1,2,3-triazol-4-yl)methyl β-D-glucopyranoside (4f)

Following the general procedure for acetate deprotection, 397 mg of **3f** and 28 mg NaOMe were stirred in 2 mL MeOH for 3.5 hours. The crude product was purified by recrystallization from acetone/hexane, yielding 128 mg (38%) of **4f** as a white solid. ^1^H NMR (MeOD, 400 MHz): δ 8.10 (s, 1H, triazole-CH), 4.97 (d, *J* = 12.5 Hz, 1H, H-1’a), 4.75 - 4.83 (m, 3H, H-1’a, H-α), 4.38 (d, *J* = 7.7 Hz, 1H, H-1), 3.89 (dd, *J* = 11.9, 2.0 Hz, 1H, H-6a), 3.67 (dd, *J* = 11.8, 5.3 Hz, 1H, H-6b), 3.26 - 3.41 (m, 3H (1H buried under solvent signal), H-3, H-4, H-5), 3.21 (dd, *J* = 8.9, 7.8 Hz, 1H, H-2), 2.95 (tt, *J* = 19.0, 7.1 Hz, 2H, H-β); ^13^C NMR (MeOD 100 MHz): δ 146.2, 126.0, 103.8, 78.2, 78.1, 75.2, 71.8, 63.1, 62.9, 43.6, 32.3; ^19^F NMR (MeOD, 282 MHz) δ -80.7, -113.68, -121.17, -122.16, -122.85, -125.61; HRESI MS calcd for C_17_H_19_N_3_O_6_ (M + H)^+^: 608.1061; found: 608.1064; Anal calcd for C_17_H_18_N_3_O_6_: C 33.62, H 2.99, N 6.92; found: C 33.62, H 2.91, N 6.85.

### (1-(3,3,4,4,5,5,6,6,7,7,8,8,9,9,10,10,10-Heptadecafluorodecyl)-1*H*-1,2,3-triazol-4-yl)methyl β-D-glucopyranoside (4g)

Following the general procedure for acetate deprotection, 425 mg (0.48 mmol) of **3g** and 28 mg (0.48 mmol) NaOMe were stirred in 2 mL MeOH for 3.5 hours. The crude product was purified by recrystallization from methanol, yielding 138 mg (41%) of **4g** as a white solid. ^1^H NMR (MeOD, 400MHz): δ (ppm) 8.11 (s, 1H), 4.98 (d, *J* = 12.5 Hz, 1H), 4.75 - 4.83 (m, 3H, H-1’a, H-α), 4.39 (d, *J* = 7.7 Hz, 1H), 3.90 (dd, *J* = 11.9, 1.8 Hz, 3H), 3.68 (dd, *J* = 11.9, 5.6 Hz, 4H), 3.25 - 3.39 (m, 3H (1H buried under solvent signal), H-3, H-4, H-5), 3.21 (dd, *J* = 9.0, 7.8 Hz, 1H), 2.95 (tt, *J* = 18.7, 7.1 Hz, 8H); ^13^C NMR (MeOD, 100 MHz): δ (ppm) 146.2, 126.0, 103.8, 78.2, 78.1, 75.2, 71.8, 63.1, 62.9, 43.6, 32.3; ^19^F NMR (282 MHz, MeOD) δ ppm -80.64, -113.67, -120.97, -121.14 (2 × CF_2_), -122.00, -122.79, -125.56 (br. s.); HRESIMS calcd for C_19_H_19_N_3_O_6_F_17_: 708.0997; found: 708.1006; Anal calcd for C_19_H_18_N_3_O_6_: C 32.26, H 2.56, N 5.94; found: 31.92, H 2.57, N 5.66.

## Cell culture experiments

### Dilutions of experimental chemical compounds

Chemical compounds stock solutions and their dilutions were prepared in dimethyl sulfoxide (DMSO; Sigma-Aldrich, St Louis, MO) and as necessary aliquots were added directly to 24- and 96-wells plates containing cells in complete media.

### Cell line & culture conditions

The human acute leukemia T-lymphocytes Jurkat cell line (Jurkat; ATCC, Manassas, VA) was used for the cytotoxicity assay [41]. Jurkat cells were derived from a 14 years old male donor afflicted with non-Hodgkin T-lymphoma. The culture medium for Jurkat cells was Roswell Park Memorial Institute medium (RPMI; HyClone, Logan, UT) with 10% heat inactivated fetal bovine serum (FBS; HyClone). The medium was supplemented with 100 U/mL penicillin and 100 μg/mL streptomycin (Lonza, Walkersville, MD). Cells growing exponentially around 60–75% confluence were counted and seeded into 96-well plate format (Greiner Bio-One, Monroe, NC) at a density of 25,000 cells in 100 μL culture media per well. All the incubation conditions were 37°C in a humidified 5% CO_2_ atmosphere. To guarantee high viability, cells were prepared as previously detailed [23]. All tests were assessed in quadruplicate.

### MTS colorimetric assay for cell viability

Jurkat cells were incubated with a gradient of the experimental compounds from 8 μM to 2000 μM. After 12 h of incubation, 20 μL of the MTS [3-(4,5-dimethylthiazol-2-yl)-5-(3-carboxymethoxyphenyl)-2-(4-sulfophenyl)-2H-tetrazolium] reagent (CellTiter 96 AQueousOne Solution Cell Proliferation Assay; Promega, Madison, WI) were added to each well and subsequently incubated for an additional 4 h for a total incubation period of 16 h. The colored formazan product was measured by absorbance at 490 nm with a reference wavelength of 650 nm using a microplate reader (SpectraMax 190 Absorbance Microplate Reader, Molecular Devices, Sunnyvale, CA). Control wells, containing the same volumes of culture medium and MTS reagent, were utilized to subtract background absorbance [9]. In addition, 1 mM of hydrogen peroxide (H_2_O_2_; Sigma-Aldrich, St Louis, MO) was used as a positive control for cytotoxicity. DMSO treated cells as solvent control and untreated (Unt) cells were also included in each experimental plate. Data are expressed as the cell viability percentage relative to DMSO treated control cells. Each experimental point was performed in quadruplicate to obtain the mean and standard deviation values.

Inhibitory concentration 50% (IC_50_) in μM is defined as the concentration of experimental compound required to inhibit 50% of the conversion of MTS to formazan, as compared with the absorbance produced by untreated cells after 16 h of incubation. Data derived from the MTS assay was used to determine the IC_50_. The two absorbance values closest to the 50% point were plotted with its corresponded chemical compound concentration and the equation of the regression line was utilized to calculate the IC_50_ as described previously [42].

### Annexin V/PI apoptosis/necrosis assay

The triazole-containing alkyl β-D-glucopyranosides **4d** and **4e** were selected because of their comparatively high toxicity (Table 1) to gain further insights into the mode and mechanism of cell death caused by this class of surfactants. The structural analog of **4d**, alkyl β-D-glucopyranosides C14G1, was selected as a control surfactant because its IC_50_ value is comparable to **4d** and **4e**. Briefly, Jurkat cells were seeded in a 24-well flat bottom tissue culture plate (Becton Dickinson, Franklin Lakes, NJ) at a cell density of 100,000 cells per well in 1 mL of culture media as described above. Triazole-containing alkyl β-D-glucopyranosides **4d** and **4e** and C14G1 were added to the cells at their respective IC_50_ followed by additional incubation of 16 h. The following controls were included in each experimental plate: (1) H_2_O_2_ (1 mM) was used as a positive control for apoptosis; (2) DMSO (1% v/v) was used as a solvent control; and (3) untreated (Unt) cells that were not exposed to DMSO or compound. All treatments including controls were run in quadruplicates. Cells from each individual well were collected in a pre-chilled ice-water cytometric tube, washed and processed essentially as detailed previously [40]. Briefly, cells were stained with a solution containing a mix of Annexin V-FITC and PI in 100 μL of binding buffer (Beckman Coulter, Miami, FL). After 15 min of incubation on ice in the dark, 300 μL of ice-cold binding buffer was added to the cell suspensions and immediately examined via flow cytometry (Cytomics FC 500; Beckman Coulter, Miami, FL). The total percentage of apoptotic cells was interpreted as the sum of both early and late stages of apoptosis (Annexin V-FITC positive), bottom and top right quadrants in a flow cytometric dot plots, respectively. Cells undergoing necrosis only stain with PI and not with Annexin V-FITC. For each sample, approximately 5,000 individual events were acquired per sample and analyzed with CXP software (Beckman Coulter, Miami, FL). Prior to data acquisition, the flow cytometer was set up and calibrated utilizing unstained, single- (PI or Annexin V-FITC) and double- (PI and Annexin V-FITC) stained cells. FL1 and FL2 detectors were plotted at *x*-axis versus *y*-axis, respectively.

### Mitochondrial membrane potential (ΔΨm) polychromatic analysis

Jurkat cells, plated in a 24 well format, were treated for 6 h [39] with IC_50_ concentration values of compounds and stained with 2 μM of JC-1 (5,5',6,6'-tetrachloro-1,1',3,3'-tetraethylbenzimidazolylcarbocyanine iodide) fluorophore following the manufacturer’s instructions (MitoProbe; Life Technologies, Grand Island, NY). Cells with intact polarized mitochondria allow JC-1 aggregation that emits a red fluorescence signal; whereas cells with depolarized mitochondria result in the formation of JC-1 monomers that emit a green fluorescence signal. The same controls described in the previous section were also included in these analyses. Data acquisition and analysis was accomplished by using CXP software (Beckman Coulter). Each data point was obtained from four independent replicates.

### Live-cell detection of intracellular caspase-3 activation

Cysteine-aspartic protease-3 (caspase-3) activation was verified by using a fluorogenic NucView 488 Caspase-3/7 substrate for live cells, following the vendor’s protocol (Biotium, Hayward, CA). This substrate diffuses easily into cells with intact plasma membrane and permits the detection of caspase-3 activation in live cells. Jurkat cells were seeded on a 24-well plate format and treated with the IC_50_ concentration of experimental compounds for 6 h. Cells exhibiting a green fluorescence signal, revealing of caspase-3 activation, were monitored *via* flow cytometry (Cytomics FC500, Beckman Coulter). The same three controls were also analyzed in parallel as described in previous sections. Each data point was obtained from three replicates. Approximately 5,000 events were collected and analyzed per sample using CXP software as described above.

### Statistical analysis

Every experimental test was accomplished in quadruplicate. To denote experimental variability, all data are plotted with the standard deviation of the mean. The statistical importance of differences between two experimental samples was achieved via two-tailed paired Student's *t*-tests. To define whether comparisons of two independent samples have statistical significance, *P* < 0.01 value was considered significant.

## Conclusions

The synthetic approach employed allows the rapid synthesis of novel triazole-linked, glucose-based surfactants **4a-g** with well-defined stereochemistry at the anomeric carbon and hydrocarbon or fluorocarbon hydrophobic tails. An initial toxicity assessment revealed that selected triazole-containing alkyl β-D-glucopyranosides (**4c-e**) and the structurally related tetradecyl β-D-glucopyranoside (i.e., C14G1) cause cytotoxic effects on Jurkat cells at low micromolar concentrations. Jurkat cells treated with triazole-containing alkyl β-D-glucopyranosides **4d** and **4e** and alkyl β-D-glucopyranoside C14G1 exhibited phosphatidylserine externalization, an early biochemical event of apoptosis. Furthermore, selected compounds induced mitochondria depolarization and caspase-3 activation that are features of induction of the intrinsic apoptotic cascade. Additional studies are needed to explore the impact of triazole-containing alkyl β-D-glucopyranosides **4** and other carbohydrate surfactants to better understand the molecular mechanisms of their toxicity.

## Additional file

Additional file 1:
**The following additional data are available with the online version of this paper.** Additional data file 1 contains copies of ^1^H and ^13^C NMR spectra.
