# Supplementary material for: Cytotoxic activity of triazole-containing alkyl β-D-glucopyranosides on a human T-cell leukemia cell line
Source: Chem Cent J. 2015 Feb 1;9:3. doi: 10.1186/s13065-014-0072-1 (PMC4333309; doi:10.1186/s13065-014-0072-1)
Supplement: Additional file 1: — The following additional data are available with the online version of this paper. Additional data file 1 contains copies of 1H and 13C NMR spectra. [file 13065_2014_72_MOESM1_ESM.pdf]

# Cytotoxic activity of triazole-containing alkyl β-D-glucopyranosides on a human T-cell leukemia cell line

Additional File 1

E. Davis Oldham[a,†], Larissa M. Nunes[b,†], Armando Varela-Ramirez[b], Stephen E. Rankin[c], Barbara L. Knutson[c], Renato J. Aguilera[b,*] and Hans-Joachim Lehmler[d,*]

[a] Department of Chemistry, University of Mary Washington, 1300 College Avenue, Fredericksburg, VA 22401, USA; [b] Cytometry, Screening and Imaging Core Facility, Border Biomedical Research Center, Department of Biological Sciences, Bioscience Research Building, University of Texas at El Paso, 500 West University Ave., El Paso, TX 79968, USA; [c] Department of Chemical and Materials Engineering, University of Kentucky, Lexington, KY 40506, USA; [d] Department of Occupational and Environmental Health, The University of Iowa, UI Research Park, Iowa City, IA 52242, USA

e-mail: eoldham@umw.edu; larissa2rh@gmail.com; avarela2@utep.edu; stephen.rankin@uky.edu; bknutson@engr.uky.edu; raguilera@utep.edu; hans-joachim-lehmler@uiowa.edu.

[*]Corresponding authors:

Hans-Joachim Lehmler: e-mail: hans-joachim-lehmler@uiowa.edu

Renato J. Aguilera: e-mail raguilera@utep.edu

[†] Authors contributed equally to this work

**Table of Contents**


Atom numbering scheme of triazole-containing alkyl β-D-glucopyranosides **3** and **4** **4**

$^{1}$H NMR spectrum of triacetyl propargyl glucose (**2**) **5**

$^{13}$C NMR spectrum of triacetyl propargyl glucose (**2**) **6**

$^{1}$H NMR spectrum of (1-octyl-1*H*-1,2,3-triazol-4-yl)methyl 2,3,4-tri-*O*-acetyl-β-glucopyranoside (**3a**) **7**

$^{13}$C NMR spectrum of (1-octyl-1*H*-1,2,3-triazol-4-yl)methyl 2,3,4-tri-*O*-acetyl-β-glucopyranoside (**3a**) **8**

$^{1}$H NMR spectrum of (1-decyl-1*H*-1,2,3-triazol-4-yl)methyl 2,3,4-tri-*O*-acetyl-β-glucopyranoside (**3b**) **9**

$^{13}$C NMR spectrum of (1-decyl-1*H*-1,2,3-triazol-4-yl)methyl 2,3,4-tri-*O*-acetyl-β-glucopyranoside (**3b**) **10**

HH COSY of (1-decyl-1*H*-1,2,3-triazol-4-yl)methyl 2,3,4-tri-*O*-acetyl-β-glucopyranoside (**3b**) **11-12**

$^{1}$H NMR spectrum of (1-dodecyl-1*H*-1,2,3-triazol-4-yl)methyl 2,3,4-tri-*O*-acetyl-β-glucopyranoside (**3c**) **13**

$^{13}$C NMR spectrum of (1-dodecyl-1*H*-1,2,3-triazol-4-yl)methyl 2,3,4-tri-*O*-acetyl-β-glucopyranoside (**3c**) **14**

$^{1}$H NMR spectrum of (1-tetradecyl-1*H*-1,2,3-triazol-4-yl)methyl 2,3,4-tri-*O*-acetyl-β-glucopyranoside (**3d**) **15**

$^{13}$C NMR spectrum of (1-tetradecyl-1*H*-1,2,3-triazol-4-yl)methyl 2,3,4-tri-*O*-acetyl-β-glucopyranoside (**3d**) **16**

$^{1}$H NMR spectrum of (1-hexadecyl-1*H*-1,2,3-triazol-4-yl)methyl 2,3,4-tri-*O*-acetyl-β-glucopyranoside (**3e**) **17**

$^{13}$C NMR spectrum of (1-hexadecyl-1*H*-1,2,3-triazol-4-yl)methyl 2,3,4-tri-*O*-acetyl-β-glucopyranoside (**3e**) **18**

$^{1}$H NMR spectrum of (1-(3,3,4,4,5,5,6,6,7,7,8,8,8-tridecafluorooctyl)-1*H*-1,2,3-triazol-4-yl)methyl 2,3,4-tri-*O*-acetyl-β-glucopyranoside (**3f**) **19**

$^{13}$C NMR spectrum of (1-(3,3,4,4,5,5,6,6,7,7,8,8,8-tridecafluorooctyl)-1*H*-1,2,3-triazol-4-yl)methyl 2,3,4-tri-*O*-acetyl-β-glucopyranoside (**3f**) **20**

$^{1}$H NMR spectrum of (1-(3,3,4,4,5,5,6,6,7,7,8,8,9,9,10,10,10-heptadecafluorodecyl)-1*H*-1,2,3-triazol-4-yl)methyl 2,3,4-tri-*O*-acetyl-β-glucopyranoside (**3g**) **21**

$^{13}$C NMR spectrum of (1-(3,3,4,4,5,5,6,6,7,7,8,8,9,9,10,10,10-heptadecafluorodecyl)-1*H*-1,2,3-triazol-4-yl)methyl 2,3,4-tri-*O*-acetyl-β-glucopyranoside (**3g**) **22**

$^{1}$H NMR spectrum of (1-octyl-1*H*-1,2,3-triazol-4-yl)methyl β-D-glucopyranoside (**4a**) **23**

$^{13}$C NMR spectrum of (1-octyl-1*H*-1,2,3-triazol-4-yl)methyl β-D-glucopyranoside (**4a**) **24**

$^{1}$H NMR spectrum of (1-decyl-1*H*-1,2,3-triazol-4-yl)methyl β-D-glucopyranoside (**4b**) **25**

$^{13}$C NMR spectrum of (1-decyl-1*H*-1,2,3-triazol-4-yl)methyl β-D-glucopyranoside (**4b**) **26**

$^{1}$H NMR spectrum of (1-dodecyl-1*H*-1,2,3-triazol-4-yl)methyl β-D-glucopyranoside (**4c**) **27**

$^{13}$C NMR spectrum of (1-dodecyl-1*H*-1,2,3-triazol-4-yl)methyl β-D-glucopyranoside (**4c**) **28**

$^{1}$H NMR spectrum of (1-tetradecyl-1*H*-1,2,3-triazol-4-yl)methyl β-D-glucopyranoside (**4d**) **29**

$^{13}$C NMR spectrum of (1-tetradecyl-1*H*-1,2,3-triazol-4-yl)methyl β-D-glucopyranoside (**4d**) **30**

$^{1}$H NMR spectrum of (1-hexadecyl-1*H*-1,2,3-triazol-4-yl)methyl β-D-glucopyranoside (**4e**) **31**

$^{13}$C NMR spectrum of (1-hexadecyl-1*H*-1,2,3-triazol-4-yl)methyl β-D-glucopyranoside (**4e**) **32**

$^{1}$H NMR spectrum of (1-(3,3,4,4,5,5,6,6,7,7,8,8,8-tridecafluorooctyl)-1*H*-1,2,3-triazol-4-yl)methyl β-D-glucopyranoside (**4f**) **33**

$^{13}$C NMR spectrum of (1-(3,3,4,4,5,5,6,6,7,7,8,8,8-tridecafluorooctyl)-1*H*-1,2,3-triazol-4-yl)methyl β-D-glucopyranoside (**4f**) **34**

$^{1}$H NMR spectrum of (1-(3,3,4,4,5,5,6,6,7,7,8,8,9,9,10,10,10-heptadecafluorodecyl)-1*H*-1,2,3-triazol-4-yl)methyl β-D-glucopyranoside (**4g**) **35**

$^{13}$C NMR spectrum of (1-(3,3,4,4,5,5,6,6,7,7,8,8,9,9,10,10,10-heptadecafluorodecyl)-1*H*-1,2,3-triazol-4-yl)methyl β-D-glucopyranoside (**4g**) **36**

**Atom numbering scheme of triazole-containing alkyl β-D-glucopyranosides 3 and 4**

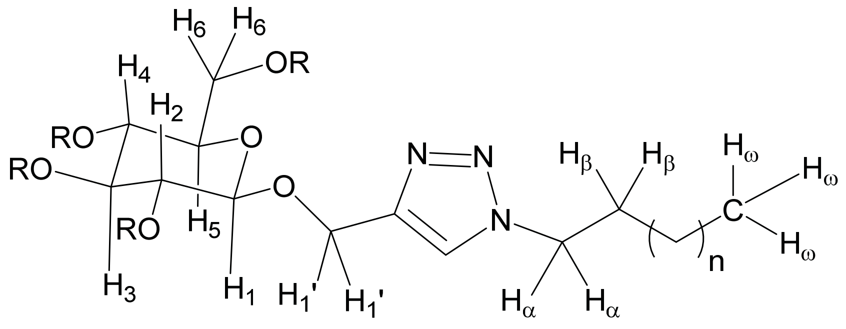


**3a-g**: R = OAc
**4a-g:** R = H

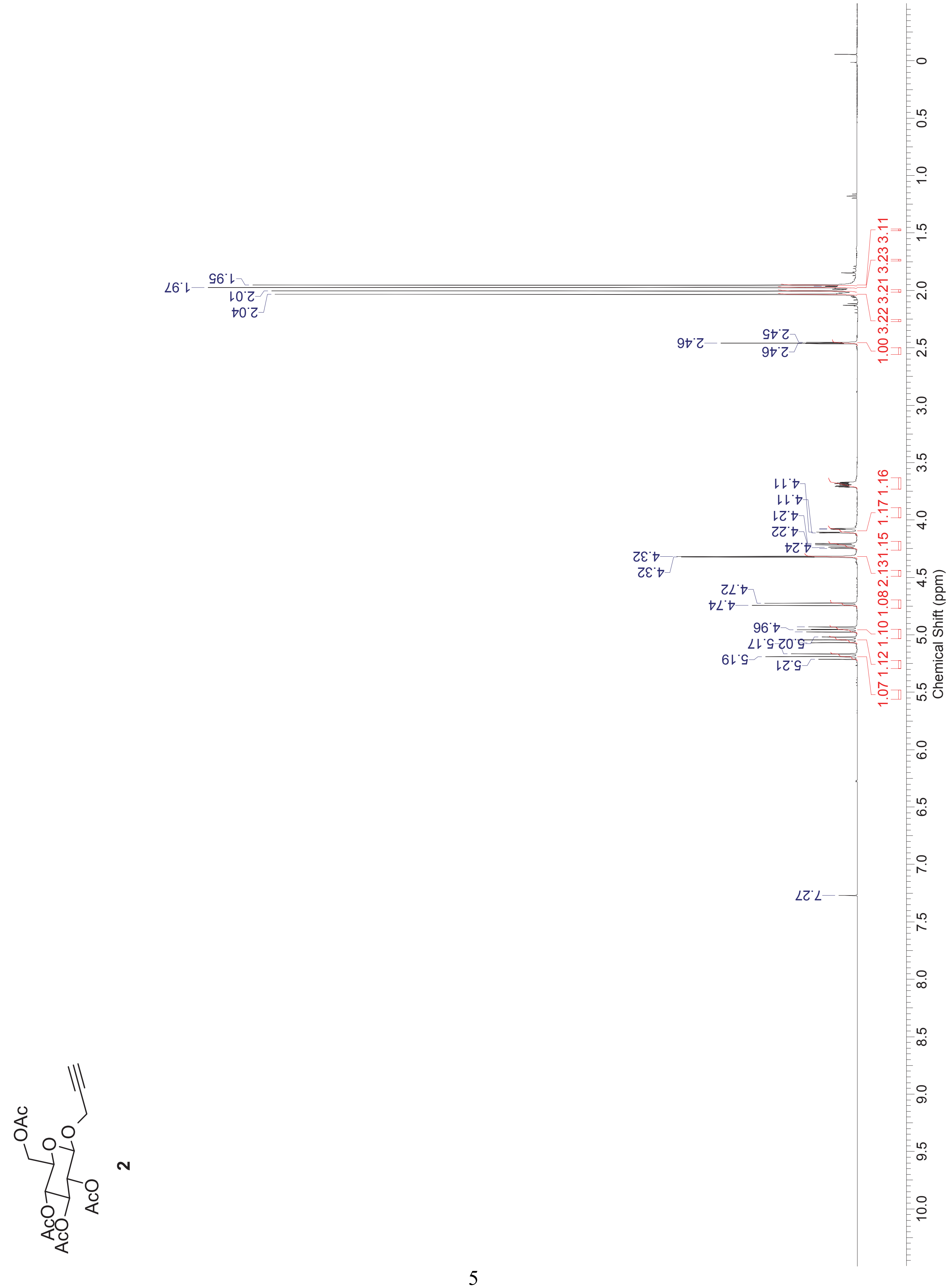
AcO
AcO
AcO
OAc
2
Chemical Shift (ppm)
1.97
1.95
2.01
2.04
2.46
2.45
2.46
4.11
4.11
4.21
4.22
4.24
4.32
4.32
4.72
4.74
4.96
5.02
5.17
5.19
5.21
7.27
1.07 1.12 1.10 1.08 2.13 1.15 1.17 1.16
1.00 3.22 3.21 3.23 3.11

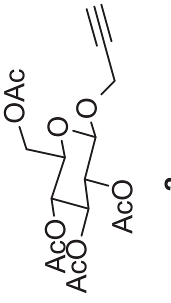

**2**

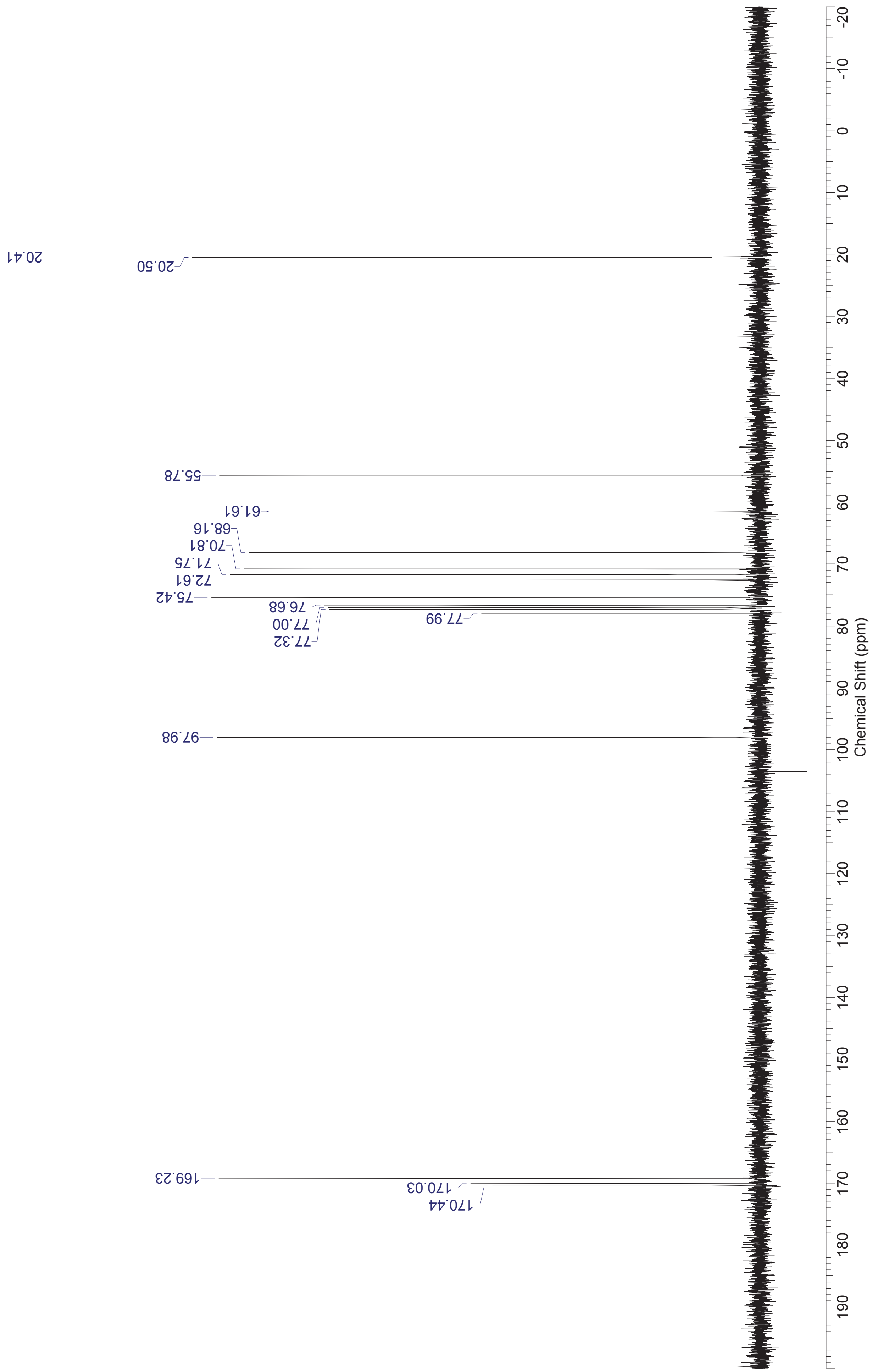
20.41
20.50
55.78
61.61
68.16
70.81
71.75
72.61
75.42
76.68
77.00
77.32
77.99
97.98
169.23
170.03
170.44
Chemical Shift (ppm)

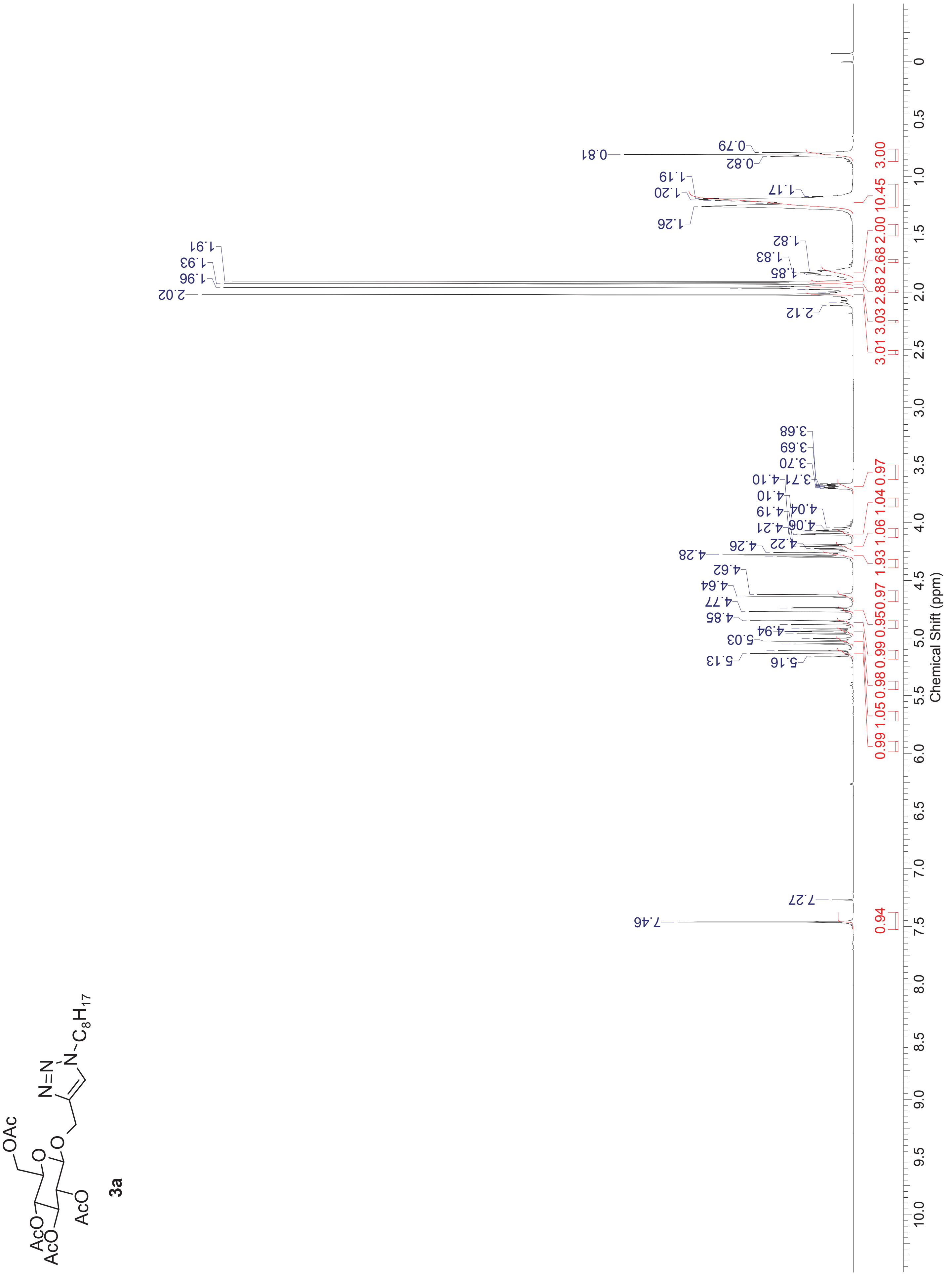


**3a**

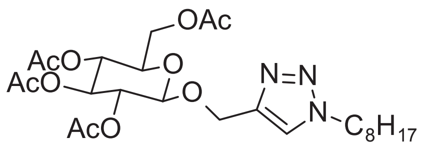

**3a**

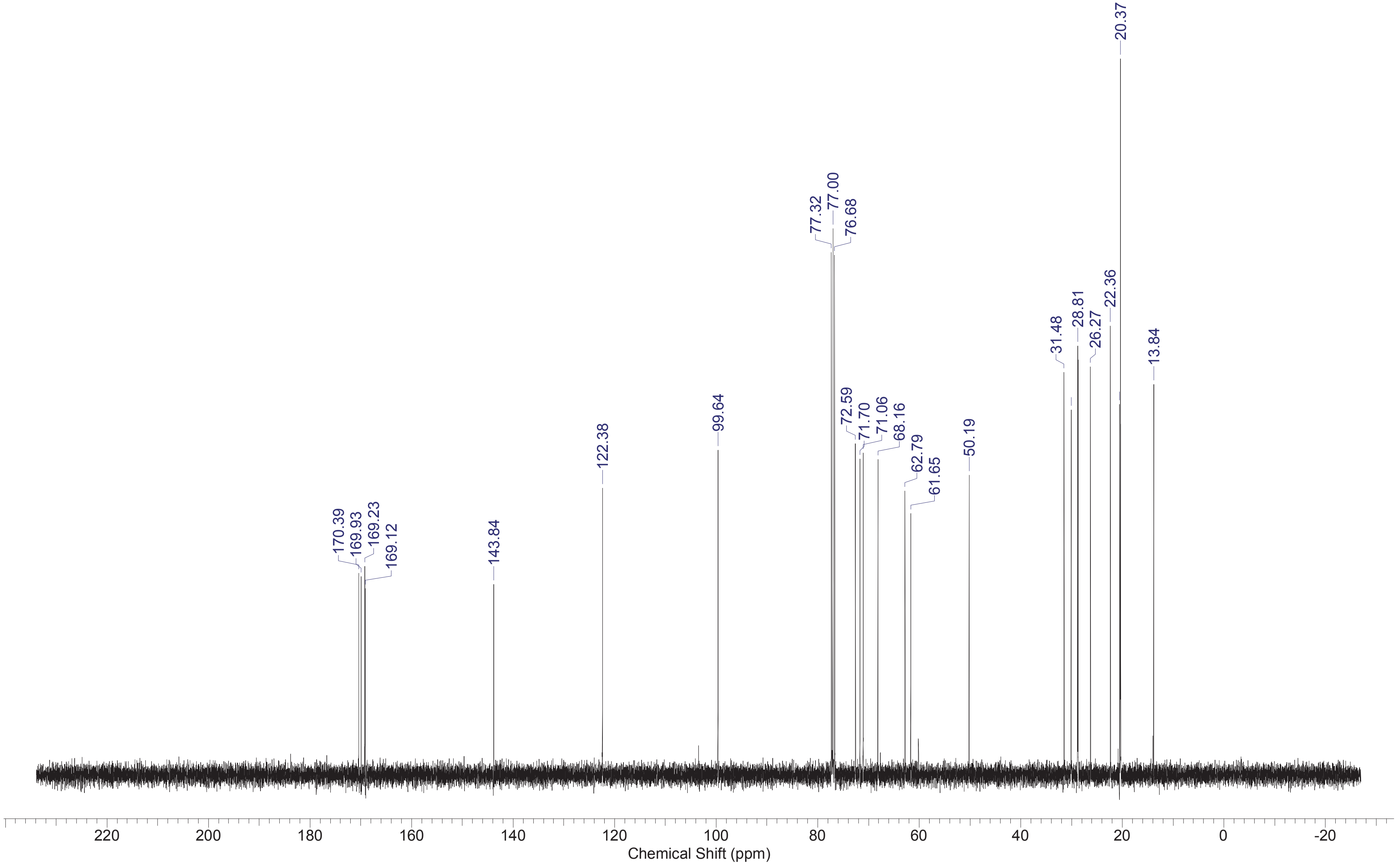

170.39
169.93
169.23
169.12
143.84
122.38
99.64
77.32
77.00
76.68
72.59
71.70
71.06
68.16
62.79
61.65
50.19
31.48
28.81
26.27
22.36
20.37
13.84
220 200 180 160 140 120 100 80 60 40 20 0 -20
Chemical Shift (ppm)

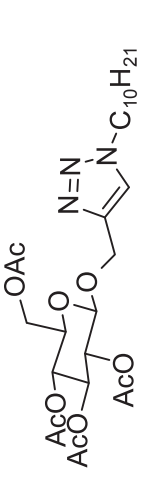
OAc
AcO
AcO
AcO
O
O
N=N
N-C10H21

**3b**

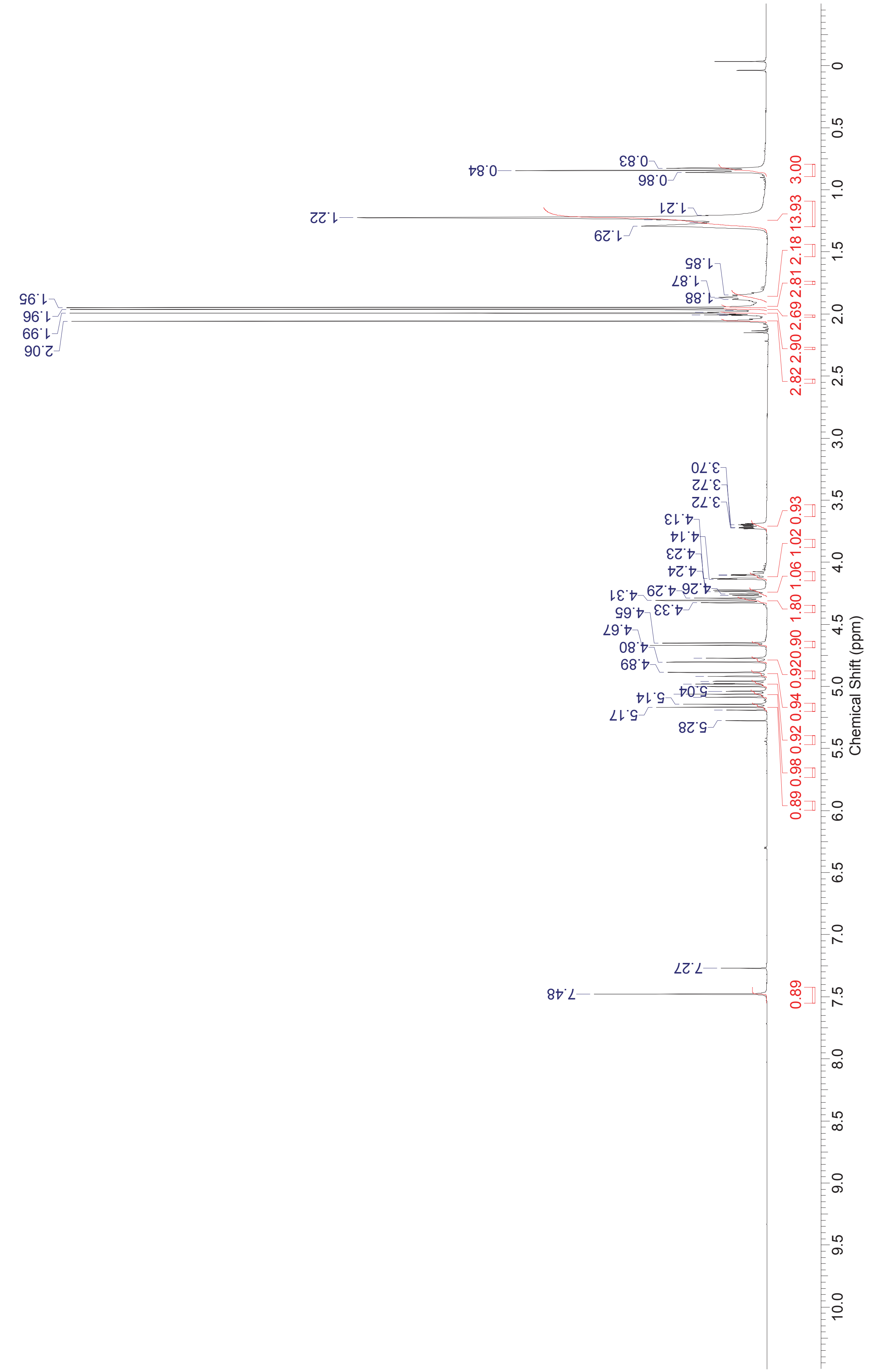
Chemical Shift (ppm)
0.89 0.98 0.92 0.94 0.92 0.92 0.90 1.80 1.06 1.02 0.93
2.82 2.90 2.69 2.81 2.18 13.93 3.00
0.89

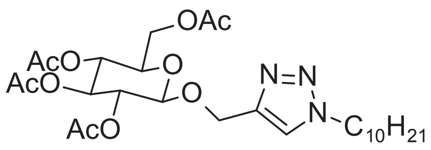
OAc
AcO
AcO
O
O
N=N
N
$C_{10}H_{21}$
AcO

**3b**

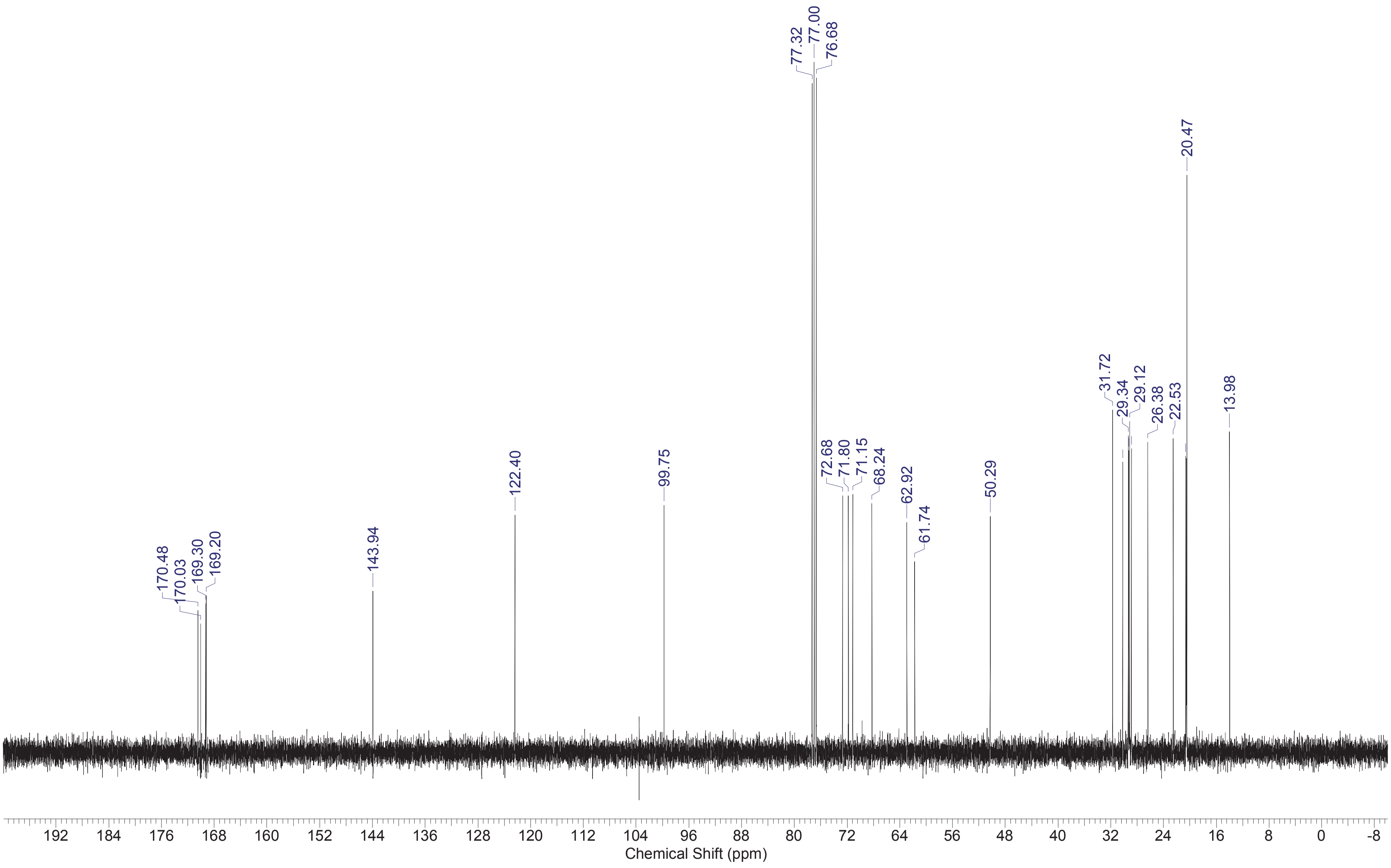
77.32
77.00
76.68
20.47
31.72
29.34
29.12
26.38
22.53
13.98
72.68
71.80
71.15
68.24
62.92
61.74
50.29
122.40
99.75
143.94
170.48
170.03
169.30
169.20
192
184
176
168
160
152
144
136
128
120
112
104
96
88
80
72
64
56
48
40
32
24
16
8
0
-8
Chemical Shift (ppm)

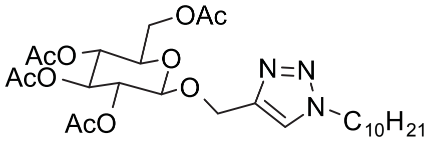
OAc
AcO
AcO
AcO
O
O
N=N
N
C10H21

**3b**

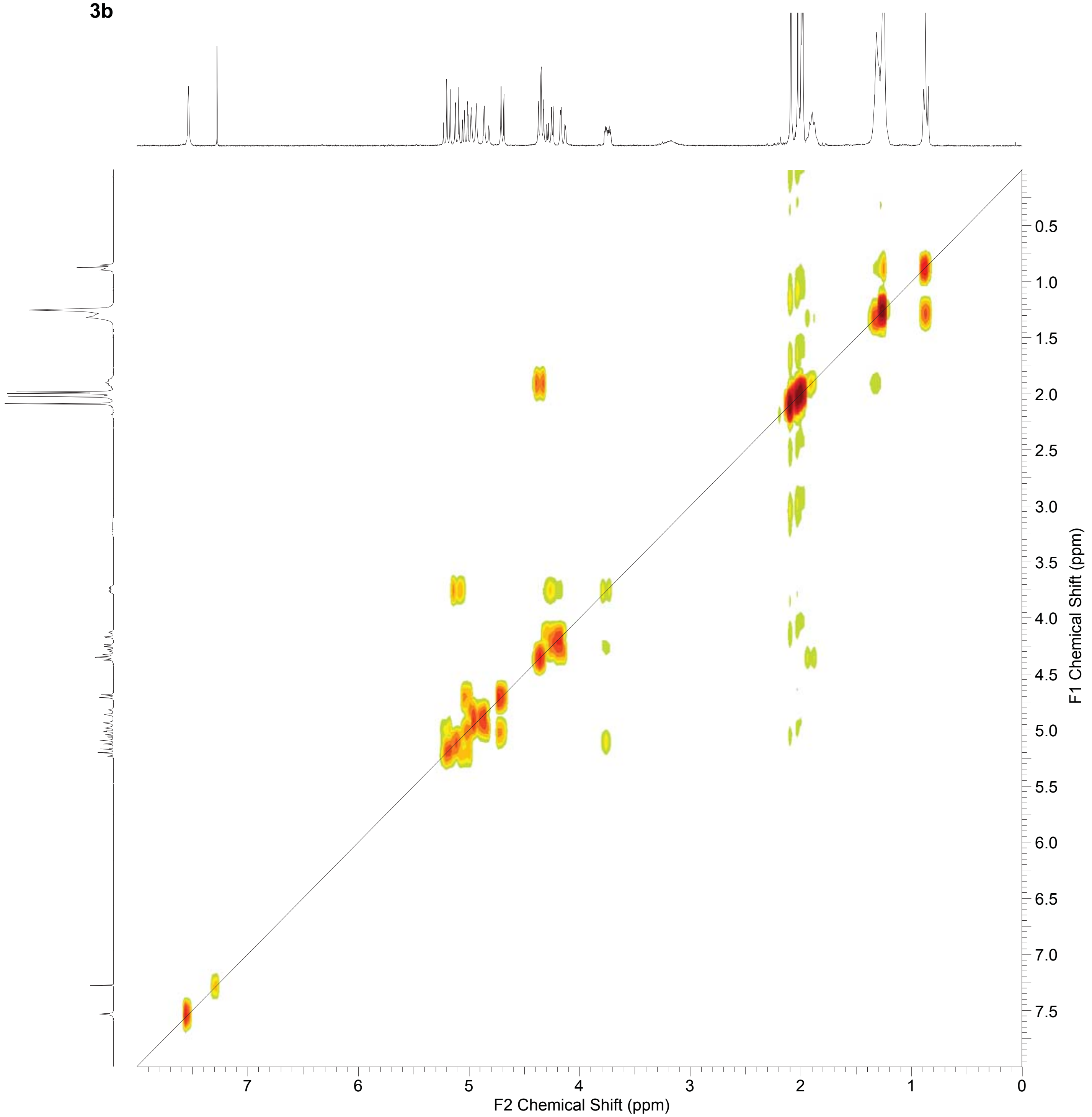
F2 Chemical Shift (ppm)
F1 Chemical Shift (ppm)

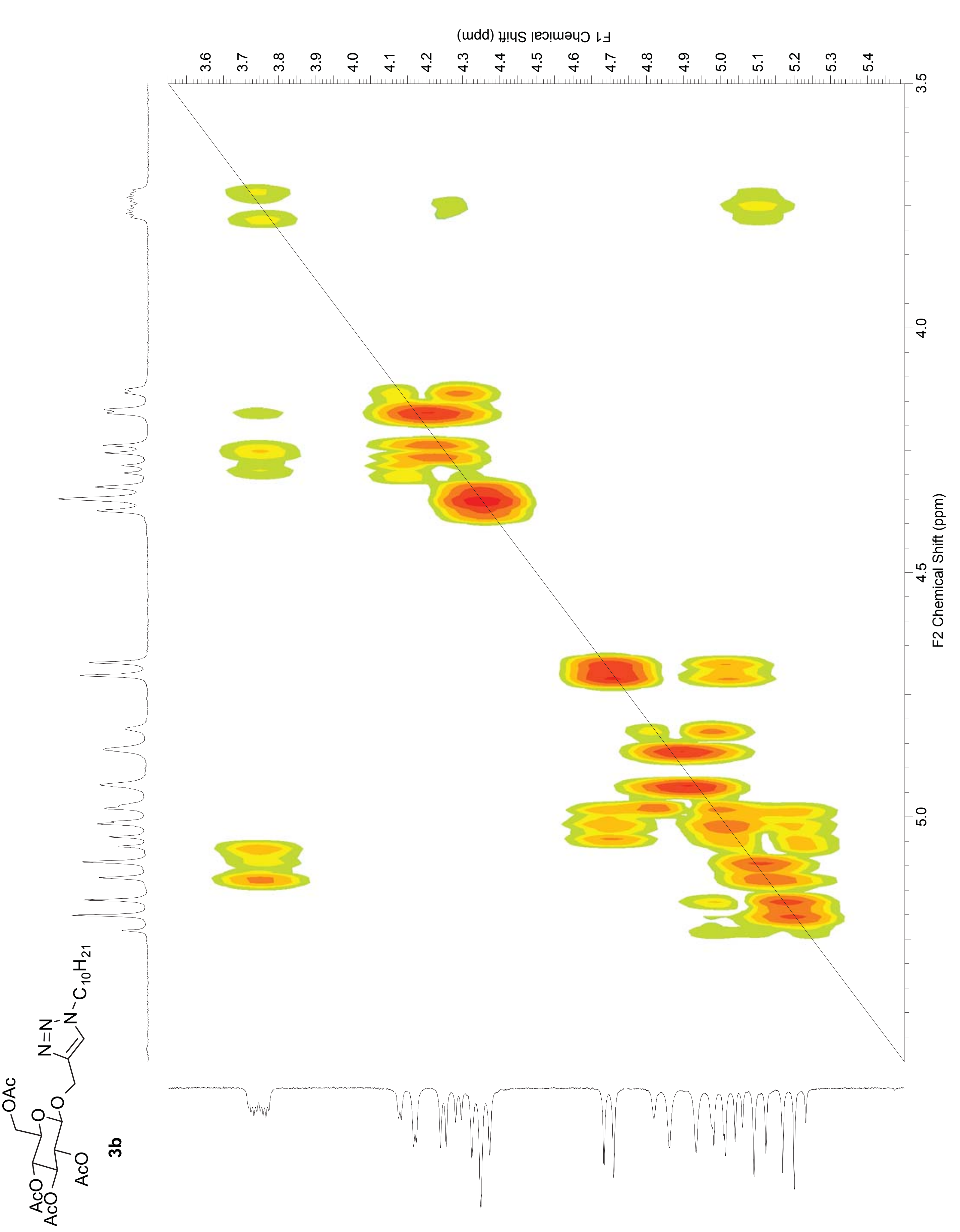
3b
F2 Chemical Shift (ppm)
F1 Chemical Shift (ppm)

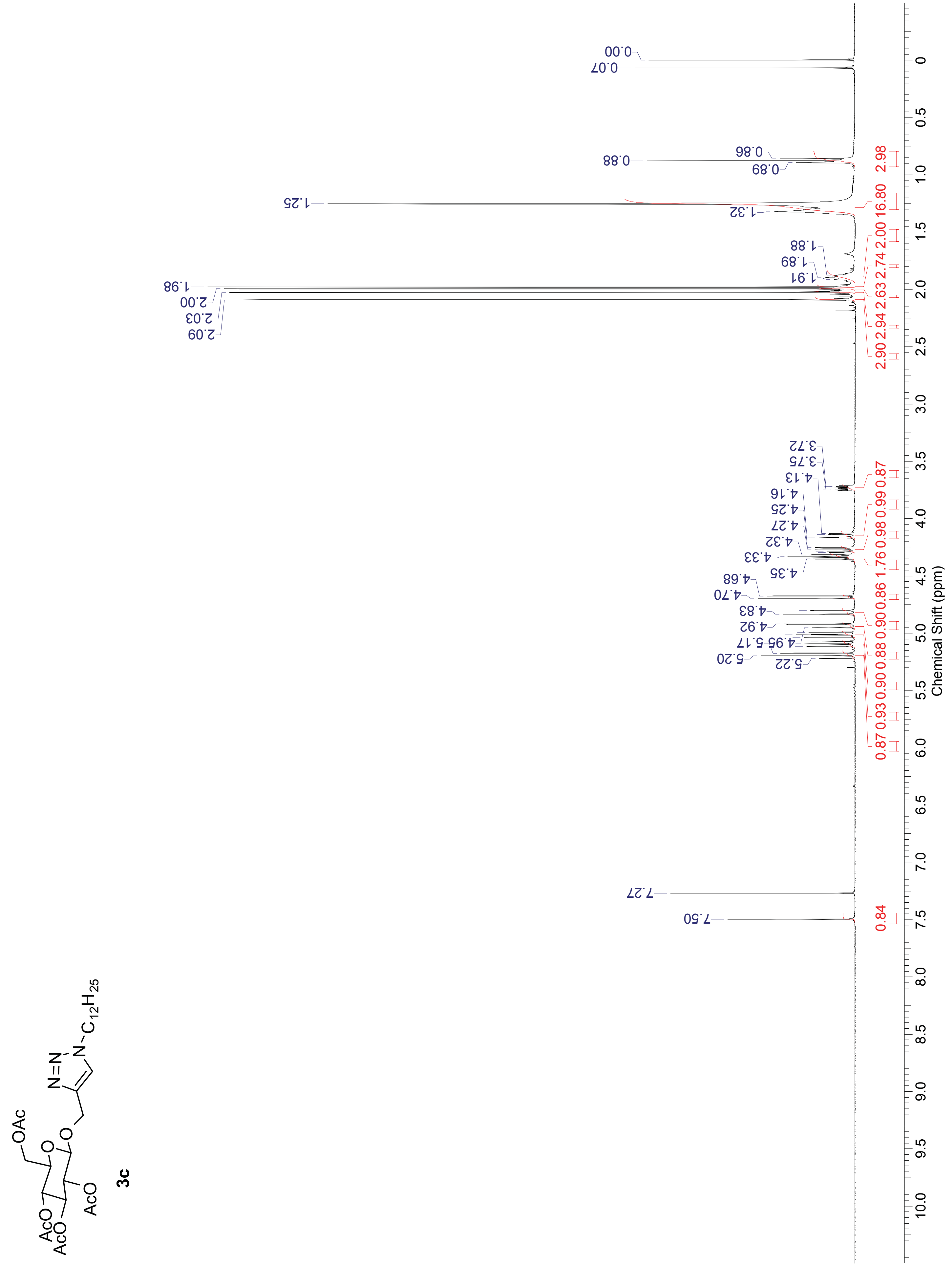

OAc
AcO
AcO
AcO
N=N
N-C12H25
0.00
0.07
0.86
0.88
0.89
1.25
1.32
1.88
1.89
1.91
1.98
2.00
2.03
2.09
3.72
3.75
4.13
4.16
4.25
4.27
4.32
4.33
4.35
4.68
4.70
4.83
4.92
4.95
5.17
5.20
5.22
7.27
7.50
2.98
16.80
2.00
2.74
2.63
2.94
2.90
0.87
0.99
0.98
1.76
0.86
0.90
0.88
0.90
0.93
0.87
0.84
Chemical Shift (ppm)


**3c**

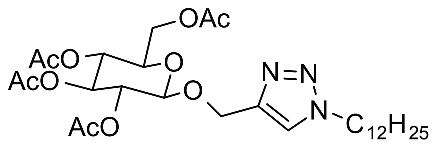

3c

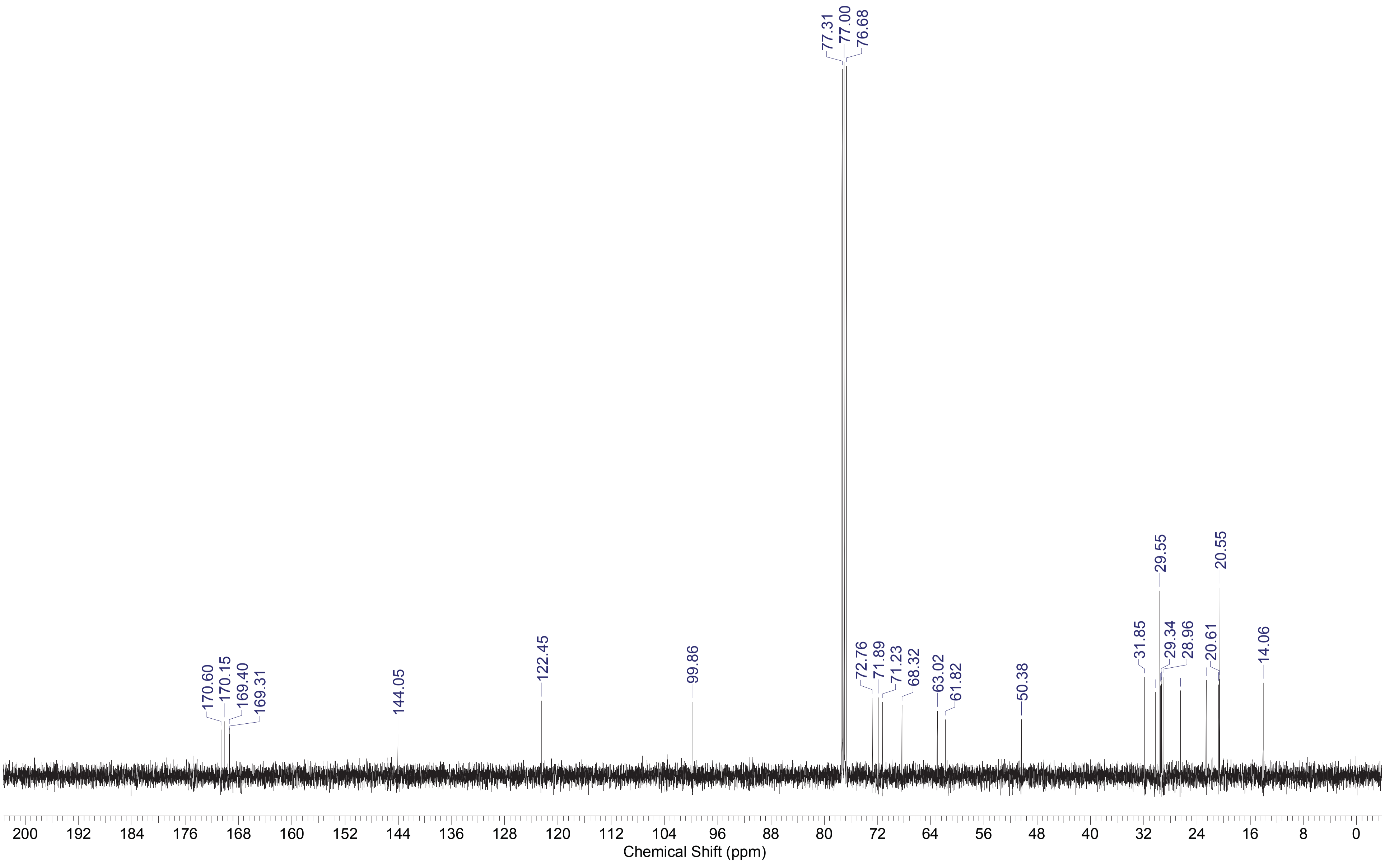

77.31
77.00
76.68
170.60
170.15
169.40
169.31
144.05
122.45
99.86
72.76
71.89
71.23
68.32
63.02
61.82
50.38
31.85
29.55
29.34
28.96
20.61
20.55
14.06
Chemical Shift (ppm)

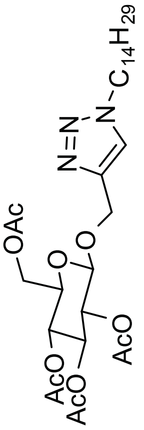


**3d**

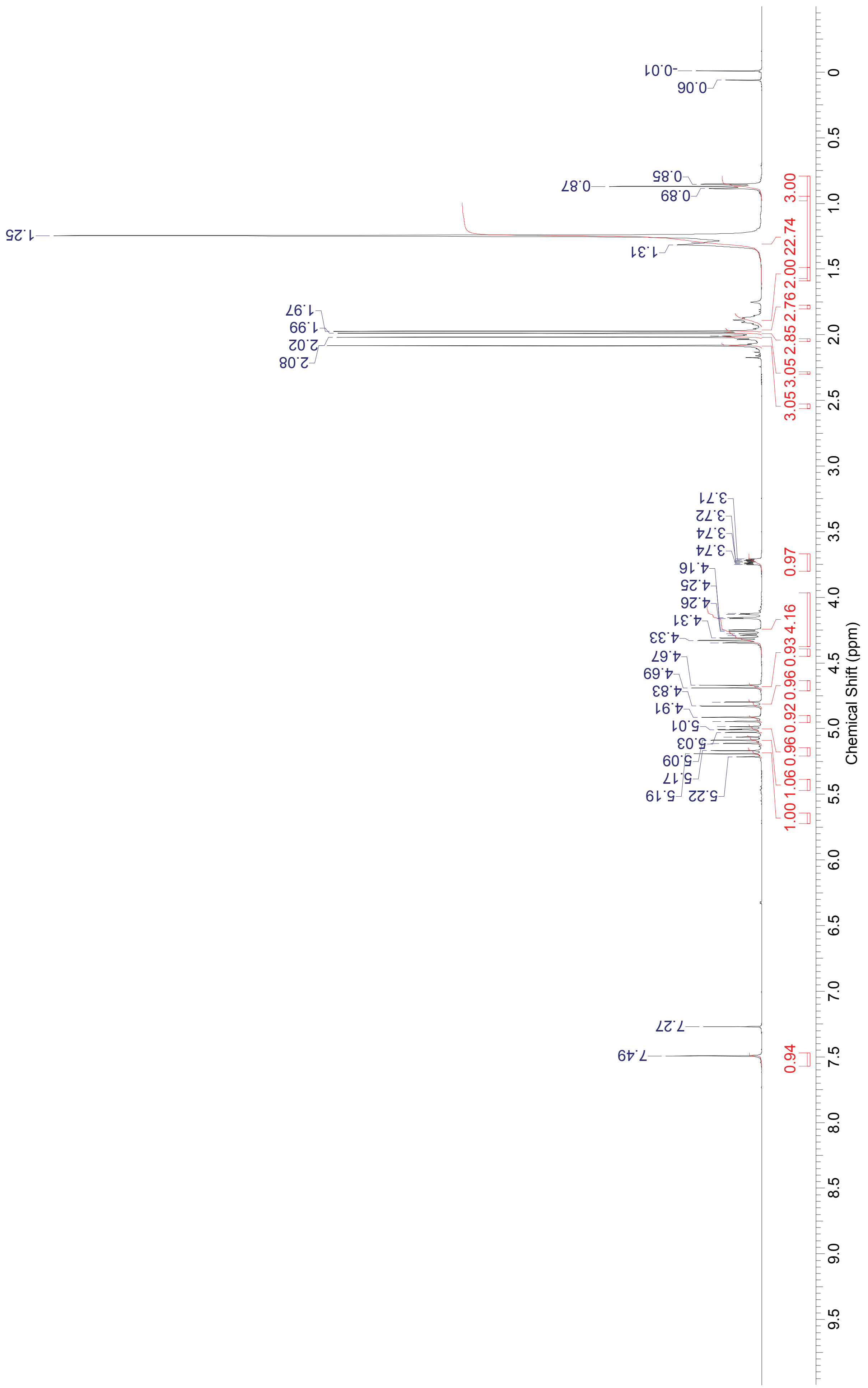

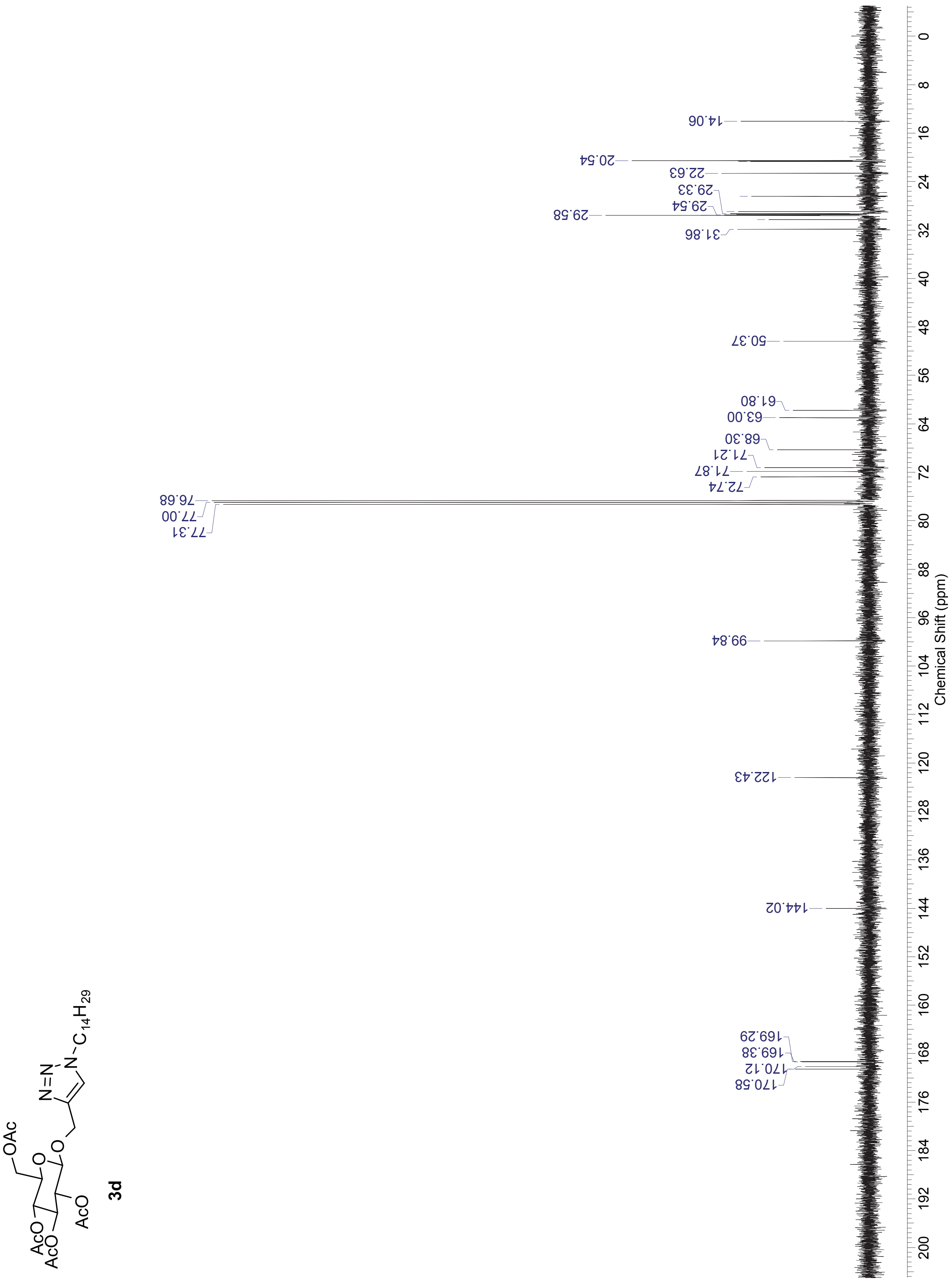
14.06
20.54
22.63
29.33
29.54
29.58
31.86
50.37
61.80
63.00
68.30
71.21
71.87
72.74
76.68
77.00
77.31
99.84
122.43
144.02
169.29
169.38
170.12
170.58
Chemical Shift (ppm)
OAc
AcO
AcO
AcO
N=N
N-$C_{14}H_{29}$

**3d**

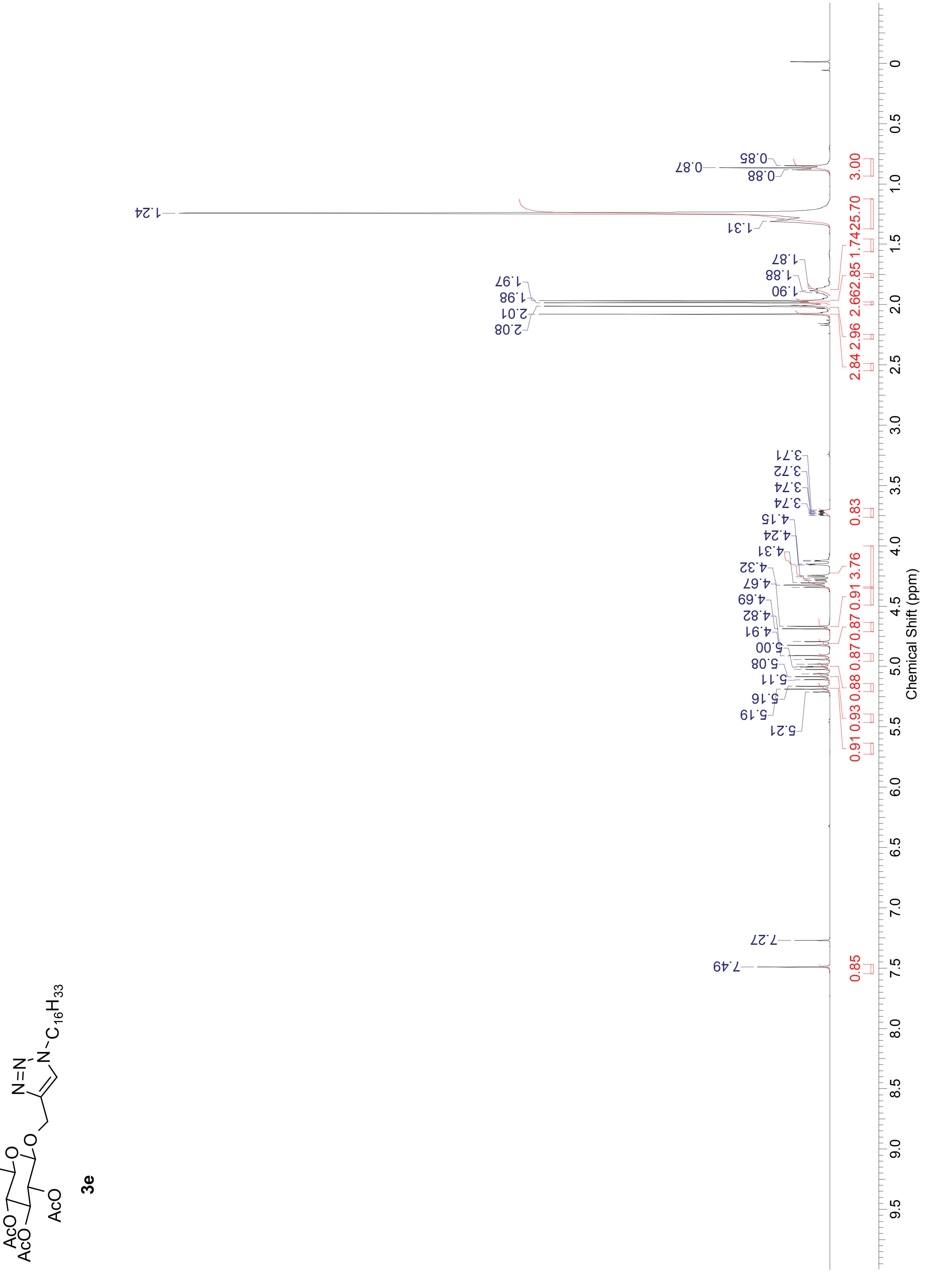
3e
Chemical Shift (ppm)

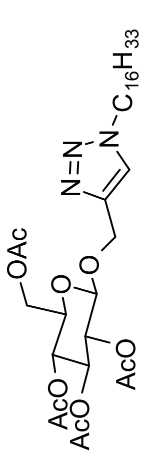
OAc
AcO
AcO
AcO
N=N
N-C16H33

**3e**

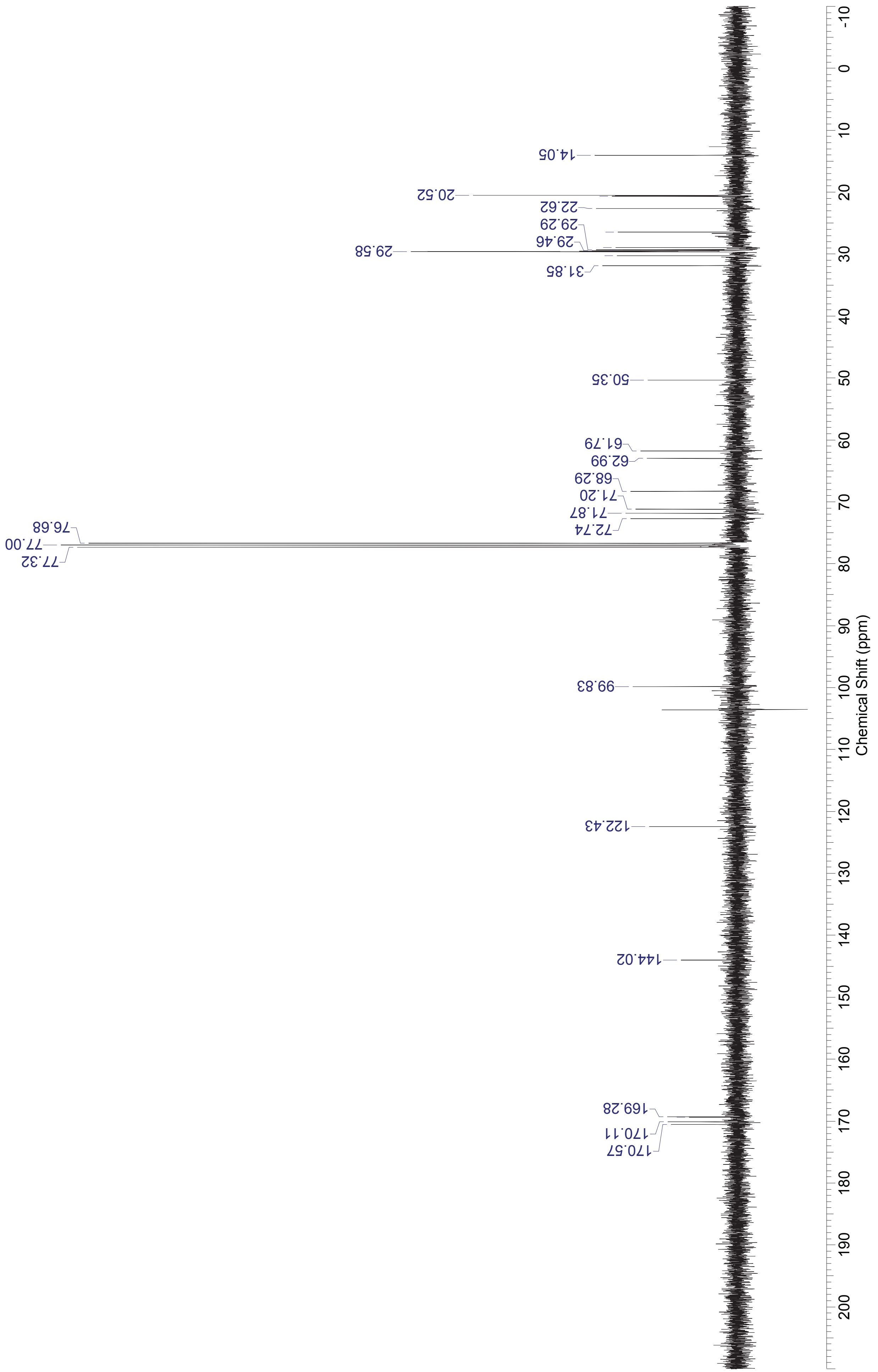
14.05
20.52
22.62
29.29
29.46
29.58
31.85
50.35
61.79
62.99
68.29
71.20
71.87
72.74
76.68
77.00
77.32
99.83
122.43
144.02
169.28
170.11
170.57
Chemical Shift (ppm)

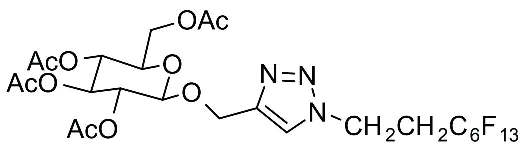
OAc
AcO
AcO
AcO
O
O
N=N
N
$CH_2CH_2C_6F_{13}$

**3f**

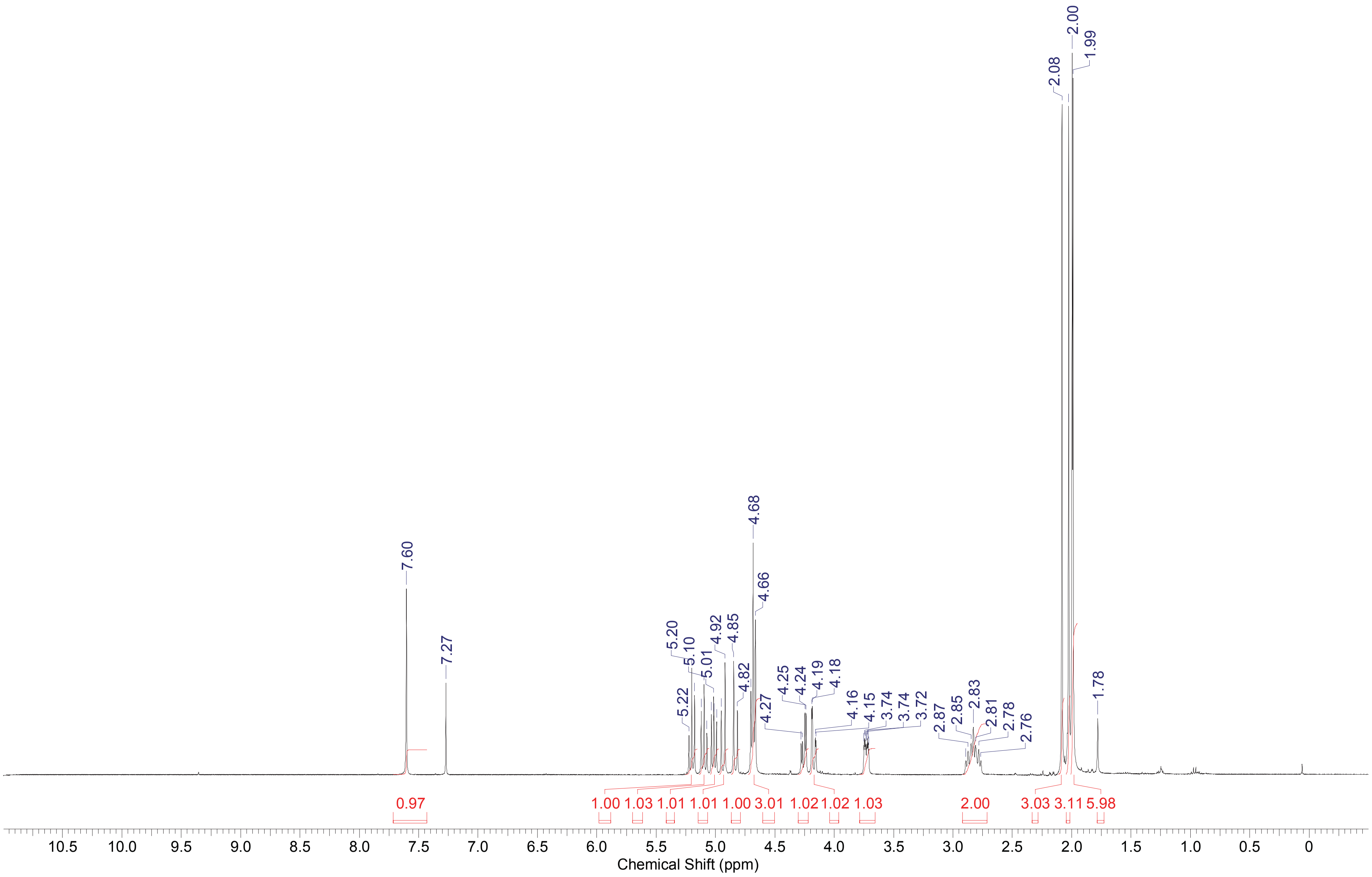
7.60
7.27
5.22
5.20
5.10
5.01
4.92
4.85
4.82
4.68
4.66
4.27
4.25
4.24
4.19
4.18
4.16
4.15
3.74
3.74
3.72
2.87
2.85
2.83
2.81
2.78
2.76
2.08
2.00
1.99
1.78
0.97
1.00
1.03
1.01
1.01
1.00
3.01
1.02
1.02
1.03
2.00
3.03
3.11
5.98
Chemical Shift (ppm)

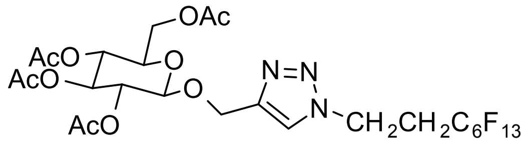
OAc
AcO
AcO
AcO
N=N
N
CH2CH2C6F13

**3f**

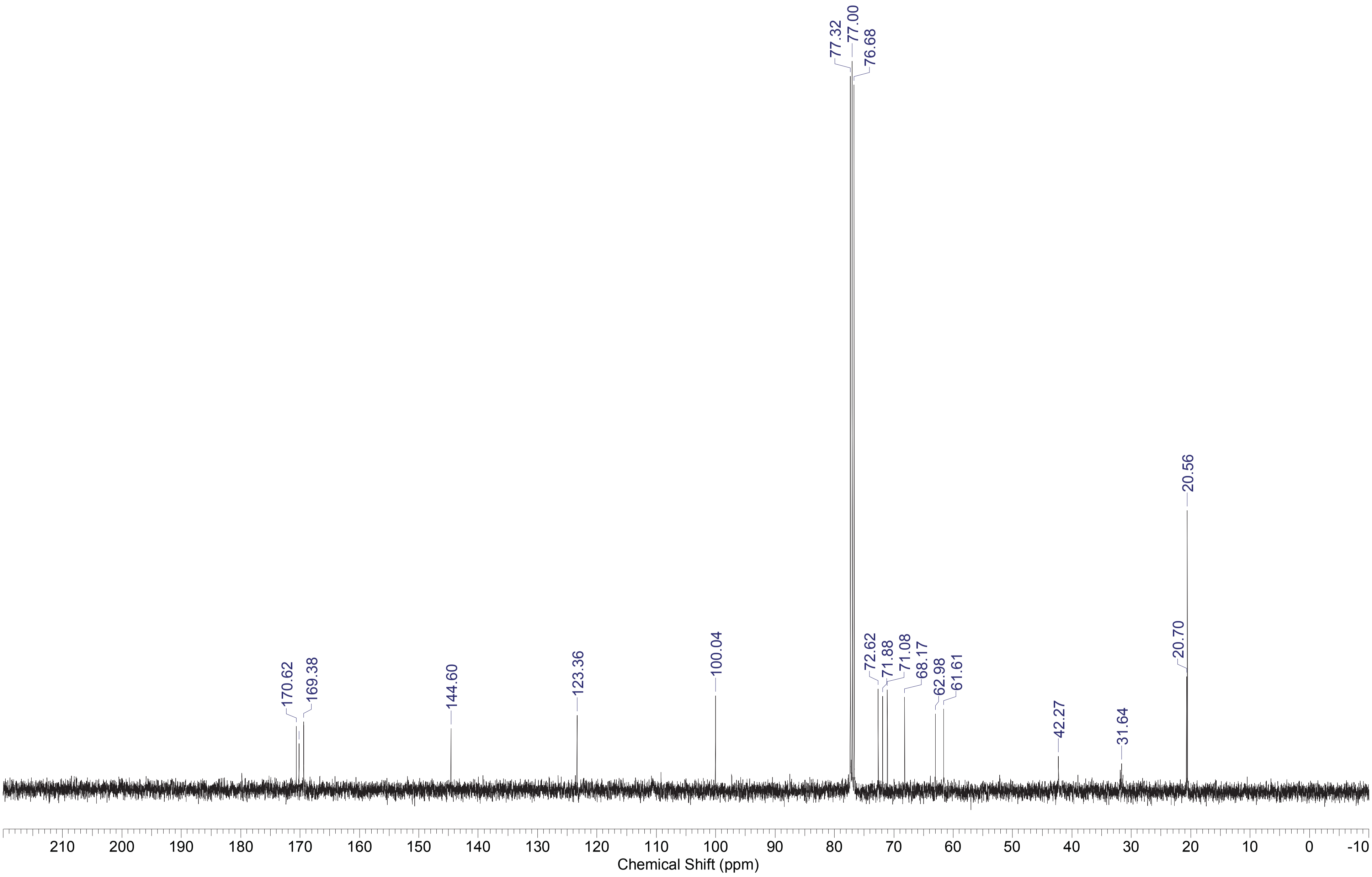
77.32
77.00
76.68
20.56
20.70
170.62
169.38
144.60
123.36
100.04
72.62
71.88
71.08
68.17
62.98
61.61
42.27
31.64
210
200
190
180
170
160
150
140
130
120
110
100
90
80
70
60
50
40
30
20
10
0
-10
Chemical Shift (ppm)

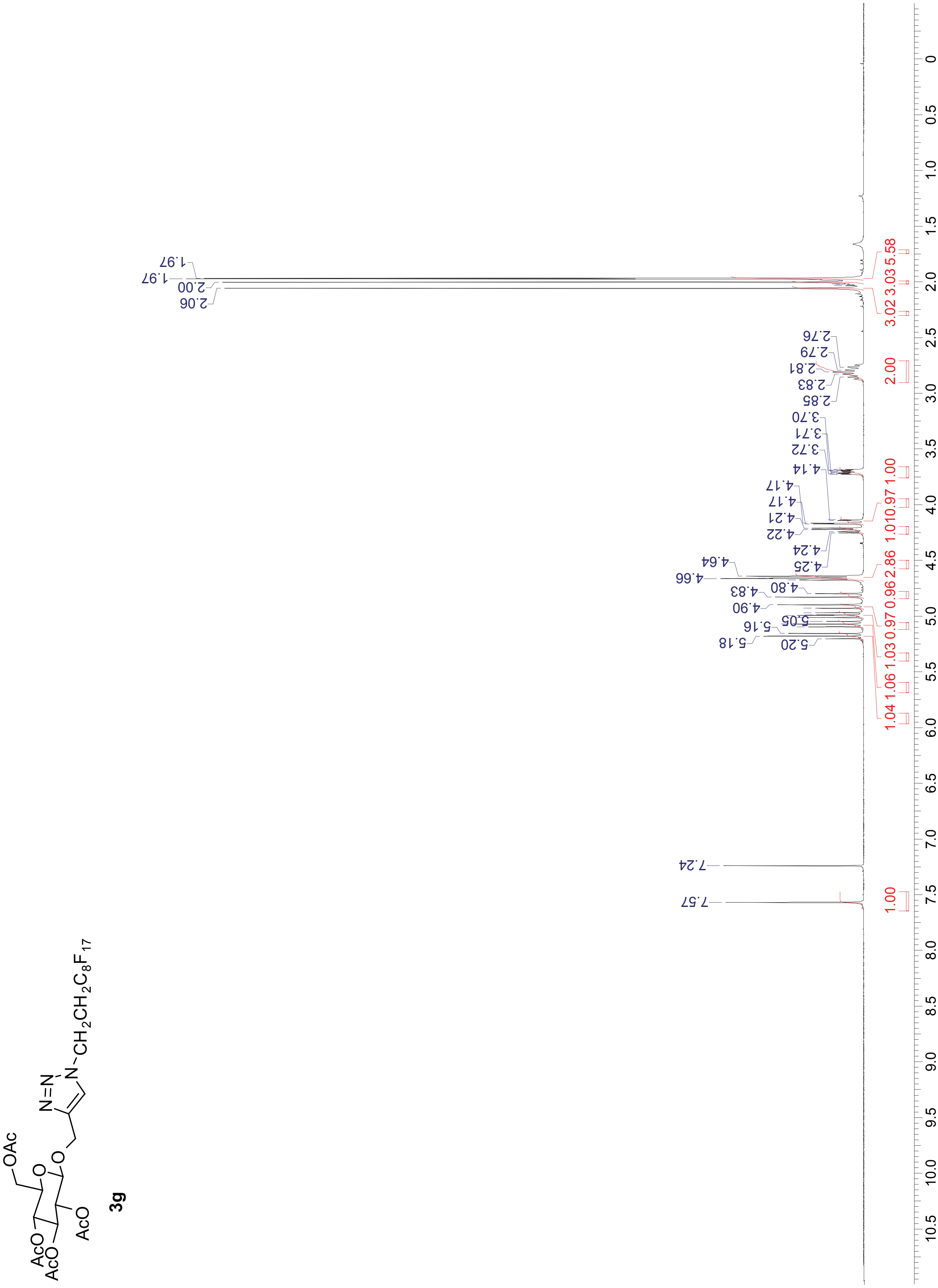
AcO
AcO
AcO
OAc
O
O
N=N
N-CH2CH2C8F17
7.57
7.24
5.20
5.18
5.16
5.05
4.90
4.83
4.80
4.66
4.64
4.25
4.24
4.22
4.21
4.17
4.17
4.14
3.72
3.71
3.70
2.85
2.83
2.81
2.79
2.76
2.06
2.00
1.97
1.97
1.00
1.04
1.06
1.03
0.97
0.96
2.86
1.01
0.97
1.00
2.00
3.02
3.03
5.58
Chemical Shift (ppm)

**3g**

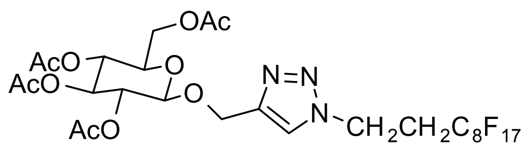
OAc
AcO
AcO
AcO
O
O
N=N
N
CH2CH2C8F17

**3g**

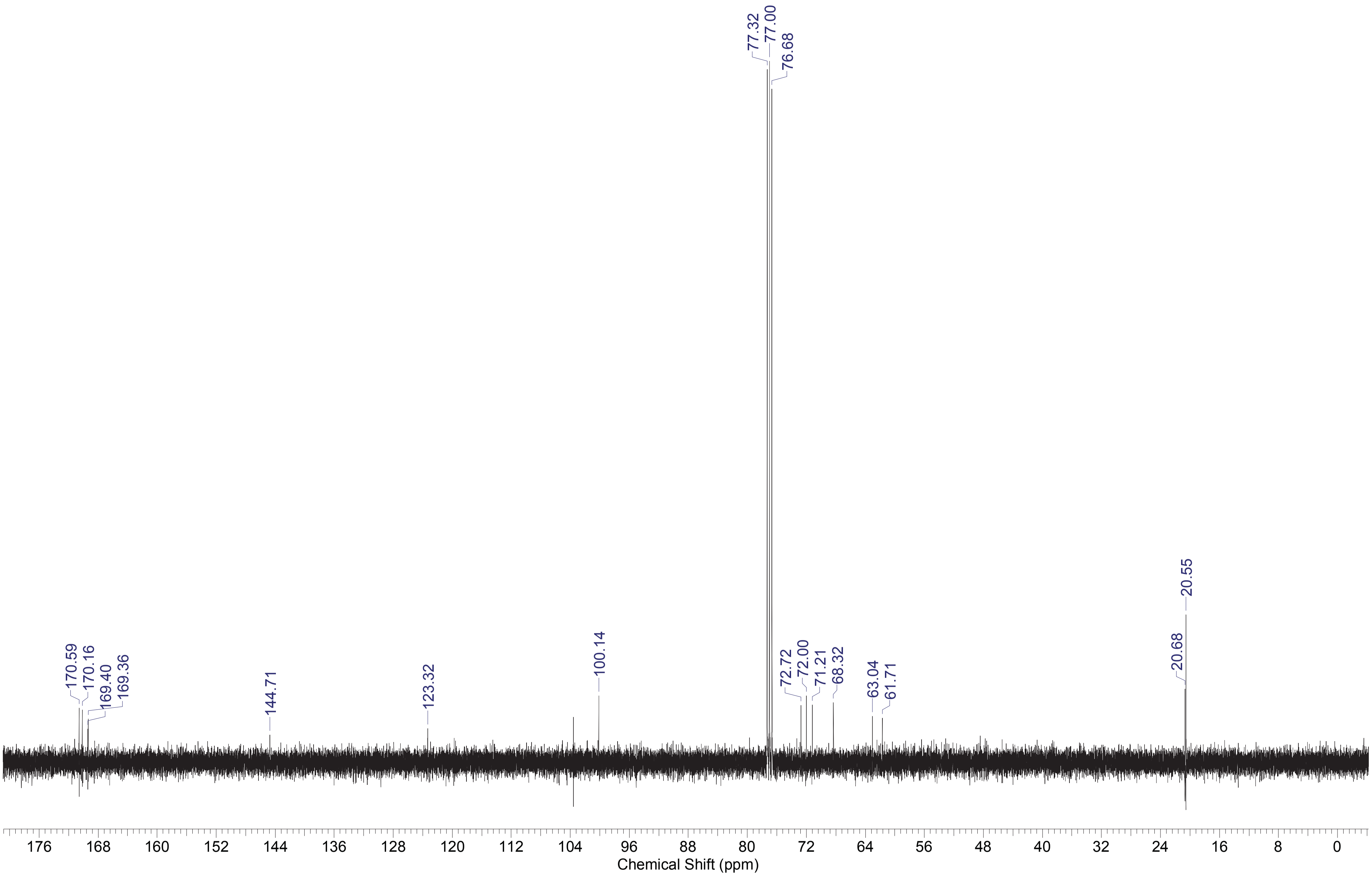
77.32
77.00
76.68
170.59
170.16
169.40
169.36
144.71
123.32
100.14
72.72
72.00
71.21
68.32
63.04
61.71
20.68
20.55
176
168
160
152
144
136
128
120
112
104
96
88
80
72
64
56
48
40
32
24
16
8
0
Chemical Shift (ppm)

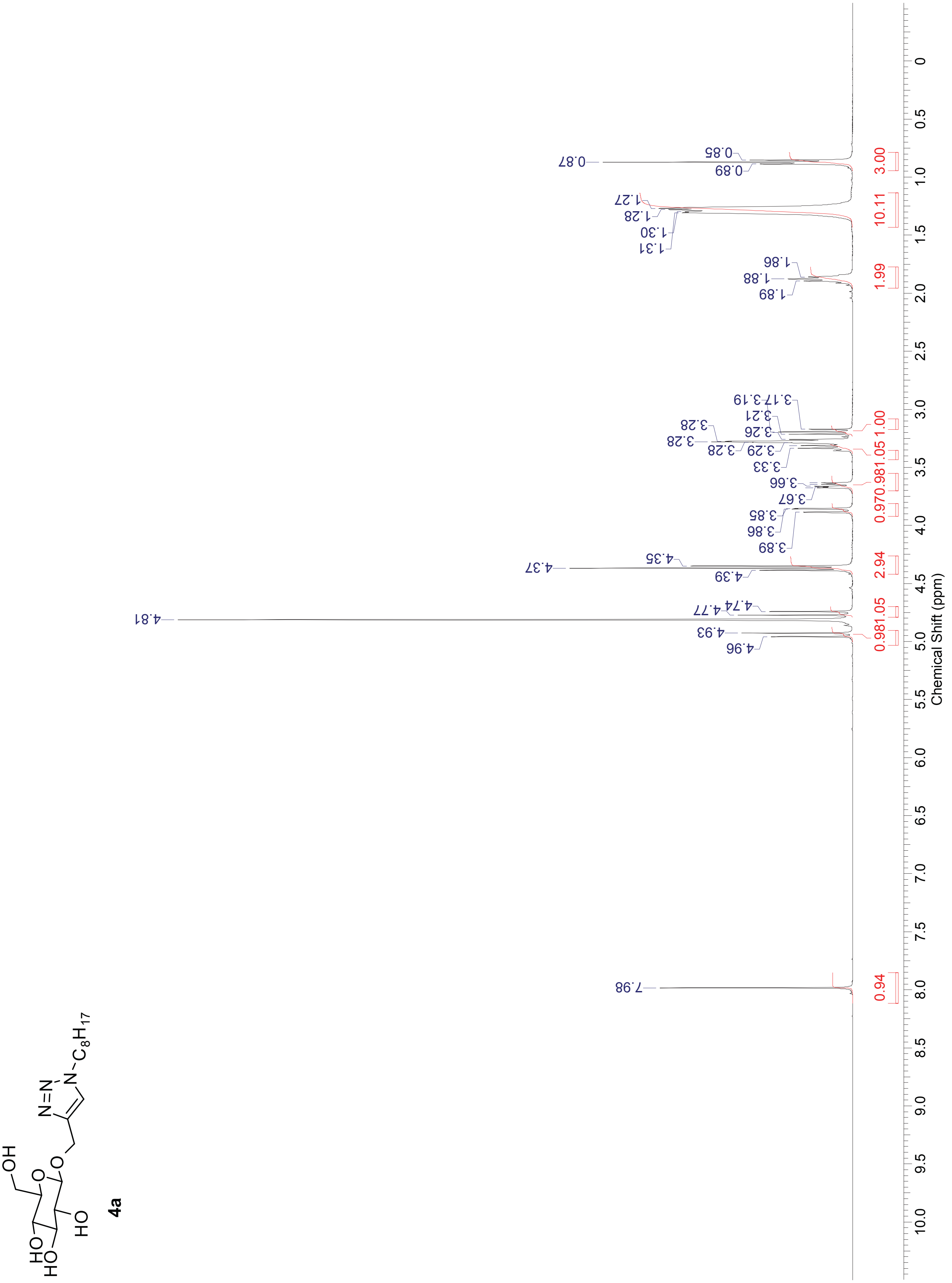

4a
N=N
N-C8H17
HO
OH
O
Chemical Shift (ppm)
0.87
0.85
0.89
1.27
1.28
1.30
1.31
1.86
1.88
1.89
3.17
3.19
3.21
3.26
3.28
3.28
3.29
3.28
3.33
3.66
3.67
3.85
3.86
3.89
4.35
4.37
4.39
4.74
4.77
4.81
4.93
4.96
7.98
3.00
10.11
1.99
1.00
1.05
0.98
0.97
2.94
1.05
0.98
0.94

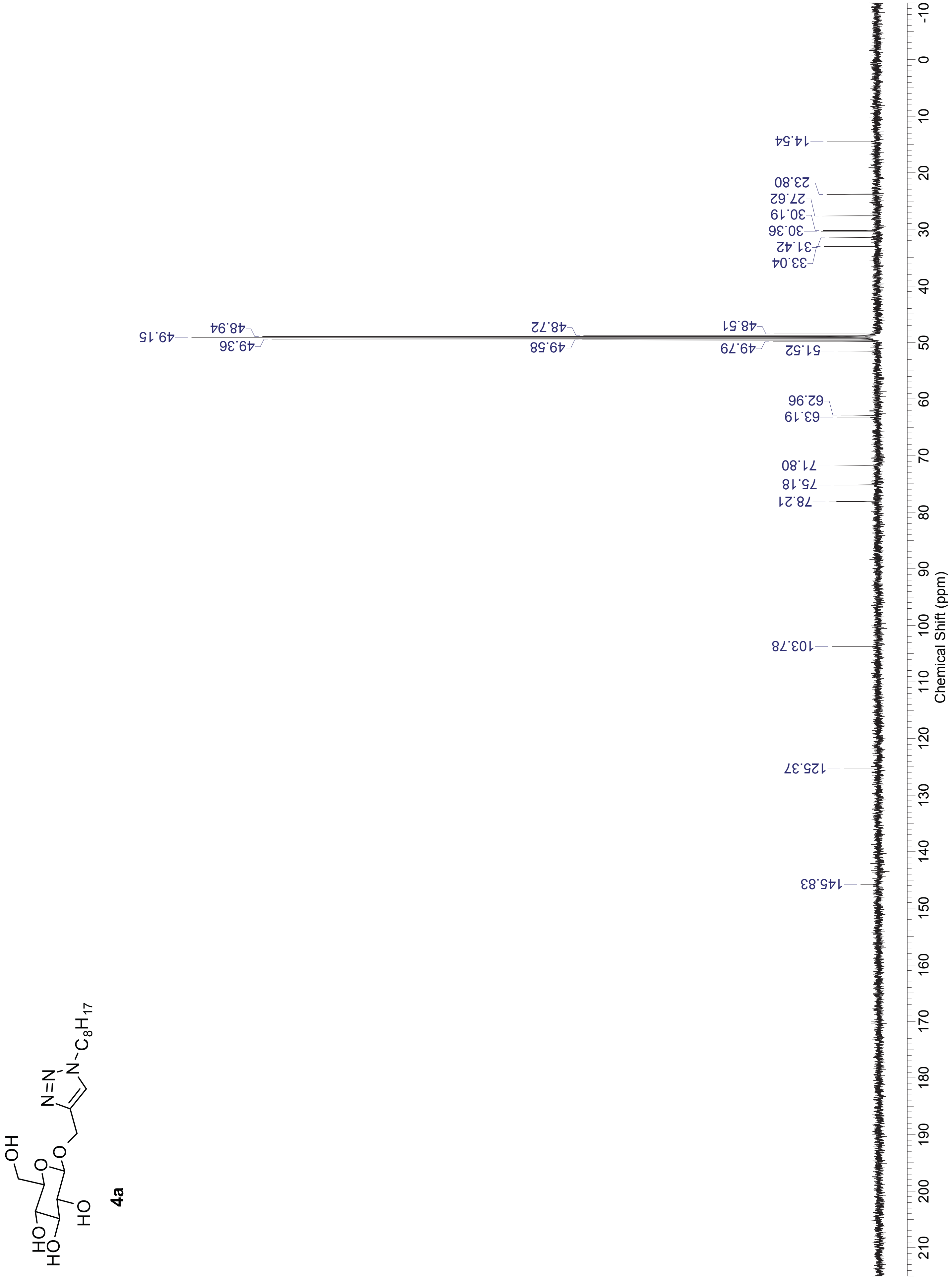
4a
14.54
23.80
27.62
30.19
30.36
31.42
33.04
48.51
48.72
48.94
49.15
49.36
49.58
49.79
51.52
62.96
63.19
71.80
75.18
78.21
103.78
125.37
145.83
Chemical Shift (ppm)

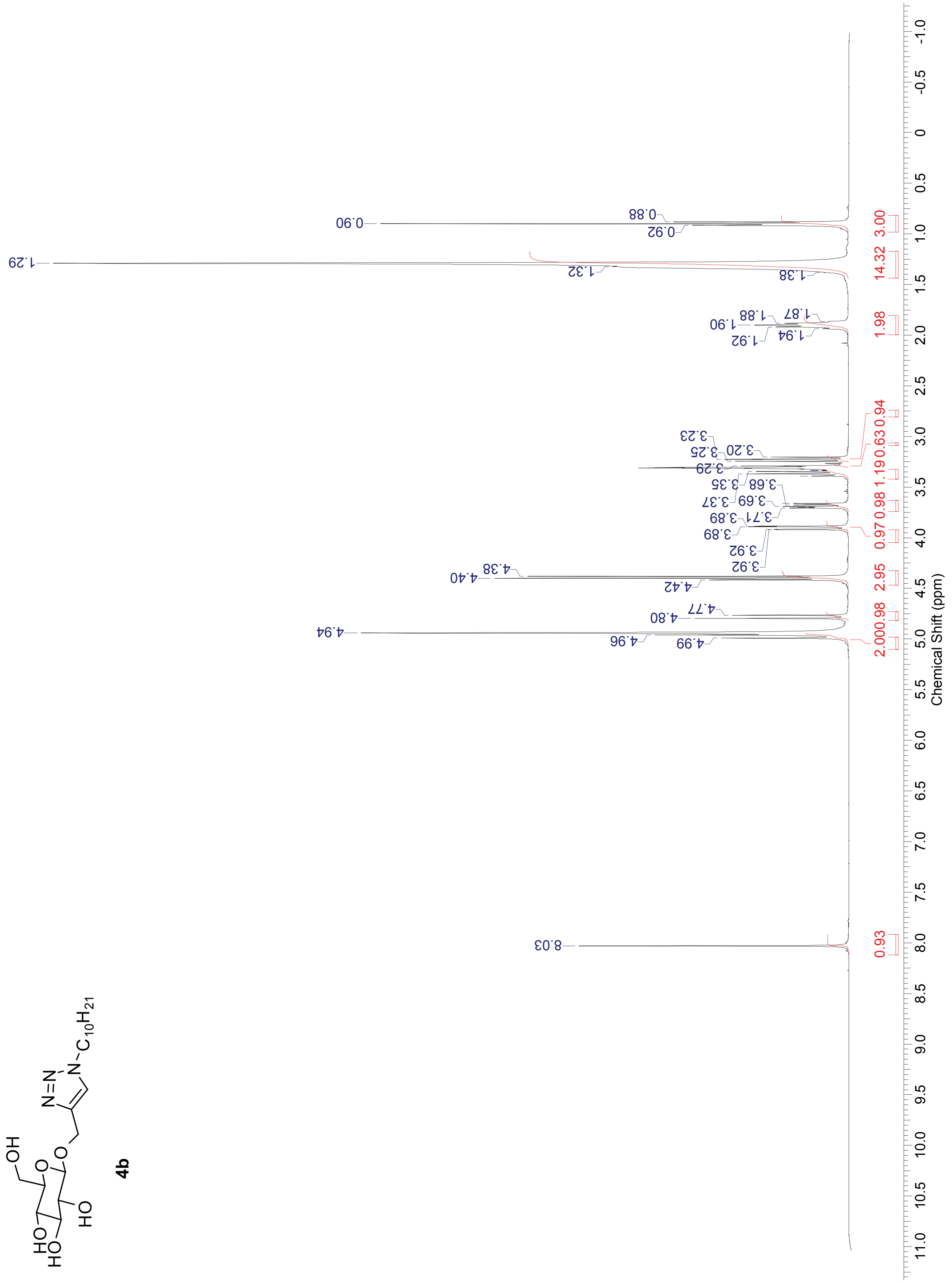
Chemical Shift (ppm)
3.00
14.32
1.98
0.94
0.63
1.19
0.98
0.97
2.95
0.98
2.00
0.93

**4b**

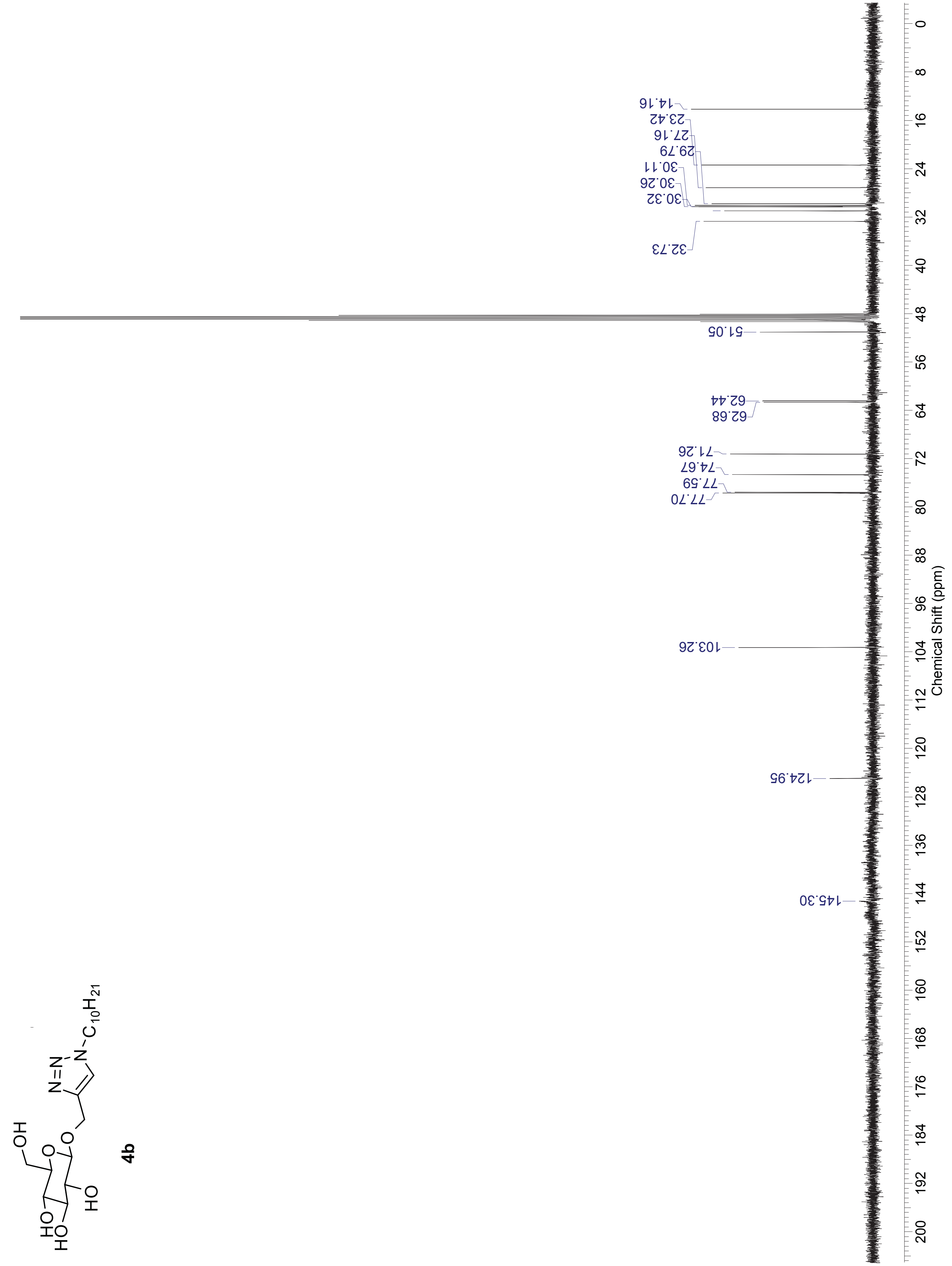
4b
N=N
N-C10H21
OH
HO
HO
HO
O
O
14.16
23.42
27.16
29.79
30.11
30.26
30.32
32.73
51.05
62.44
62.68
71.26
74.67
77.59
77.70
103.26
124.95
145.30
Chemical Shift (ppm)

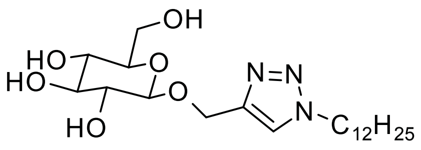

**4c**

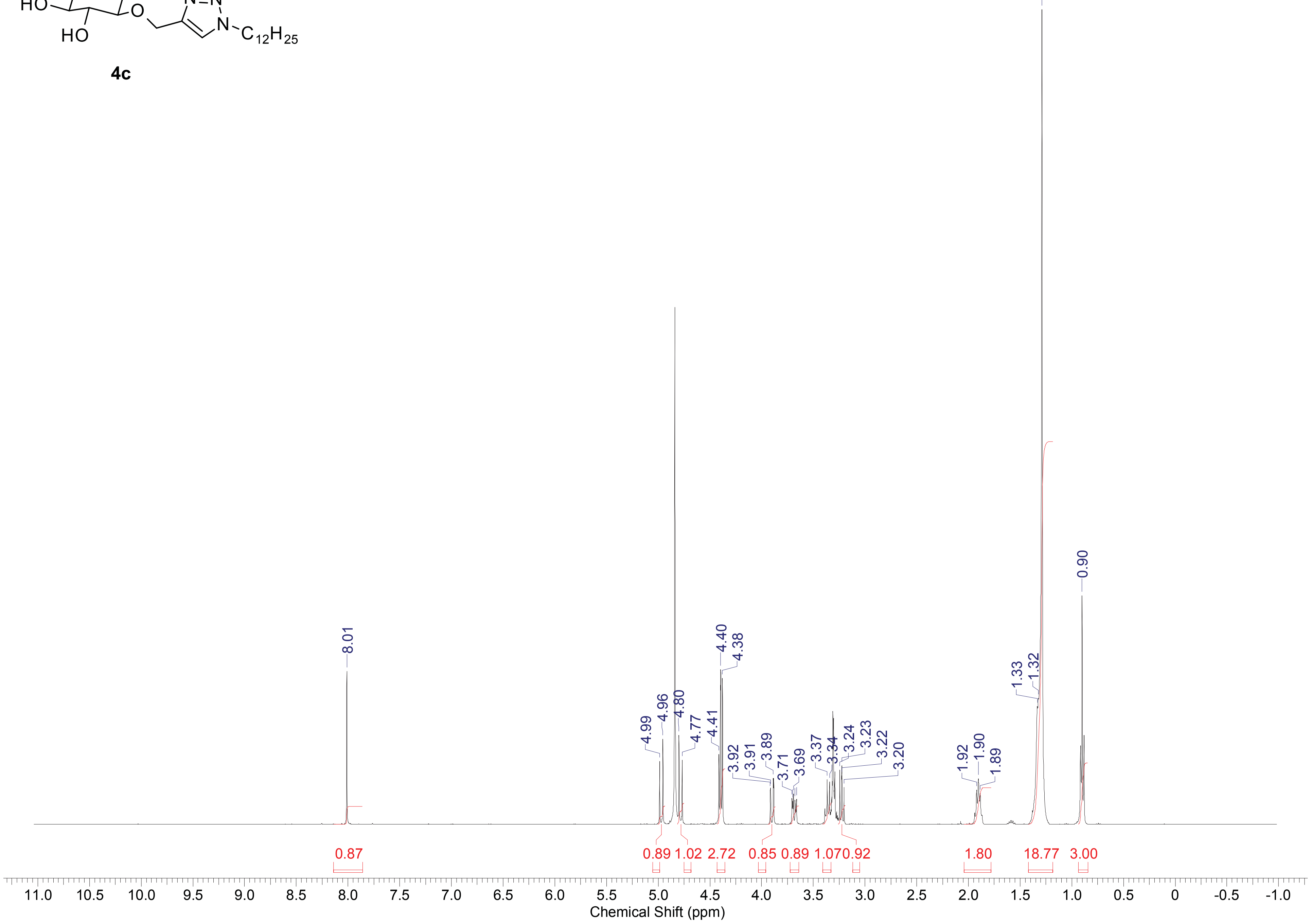

8.01
4.99
4.96
4.80
4.77
4.41
4.40
4.38
3.92
3.91
3.89
3.71
3.69
3.37
3.34
3.24
3.23
3.22
3.20
1.92
1.90
1.89
1.33
1.32
1.29
0.90
0.87
0.89
1.02
2.72
0.85
0.89
1.07
0.92
1.80
18.77
3.00
11.0 10.5 10.0 9.5 9.0 8.5 8.0 7.5 7.0 6.5 6.0 5.5 5.0 4.5 4.0 3.5 3.0 2.5 2.0 1.5 1.0 0.5 0 -0.5 -1.0
Chemical Shift (ppm)

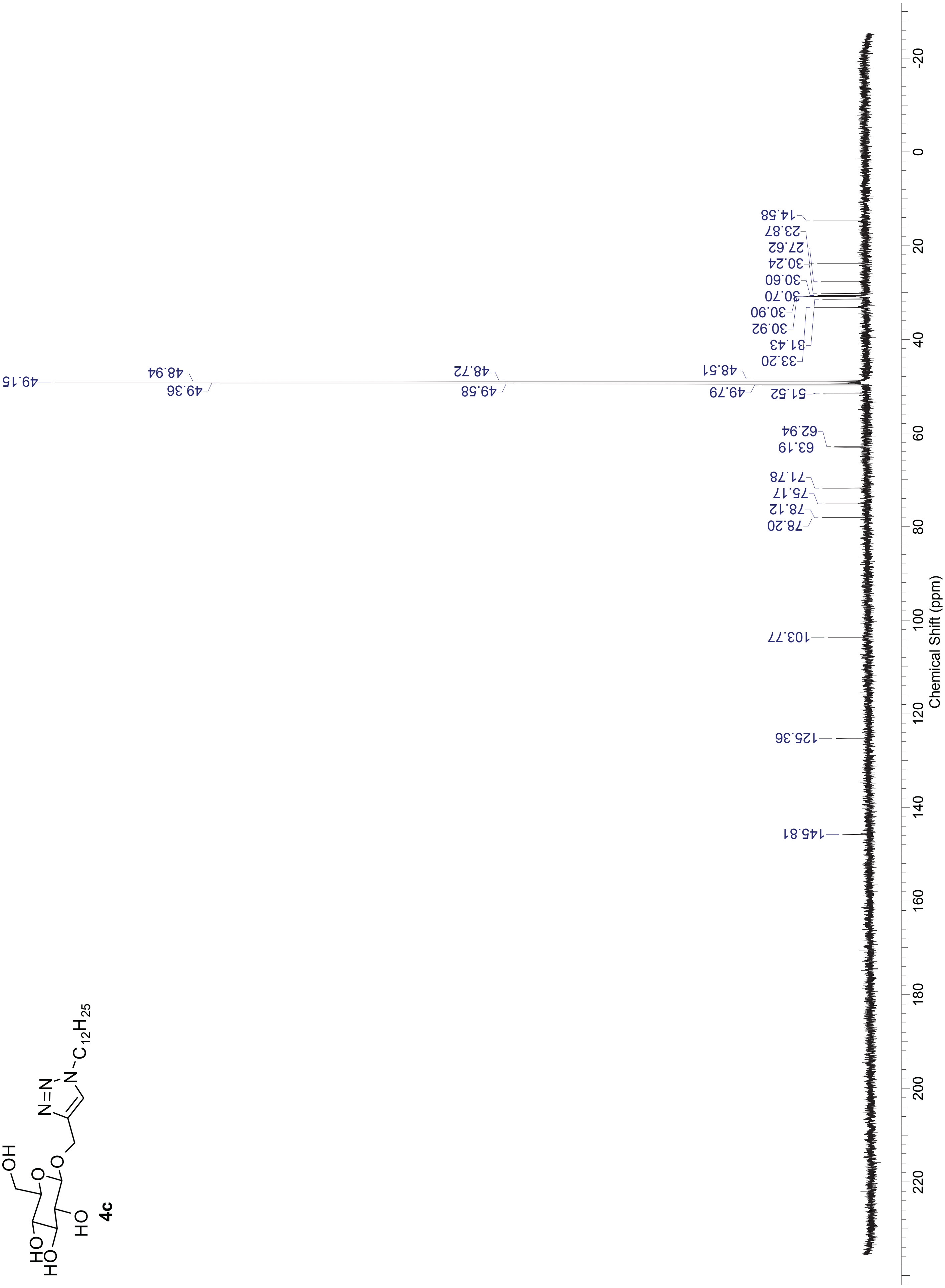

14.58
23.87
27.62
30.24
30.60
30.70
30.90
30.92
31.43
33.20
48.51
48.72
48.94
49.15
49.36
49.58
49.79
51.52
62.94
63.19
71.78
75.17
78.12
78.20
103.77
125.36
145.81
Chemical Shift (ppm)
N=N
N
$C_{12}H_{25}$
OH
O
O
HO
HO
HO

4c

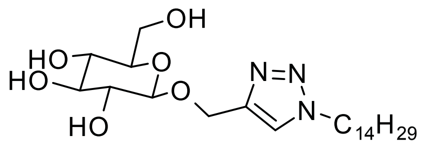
OH
HO
HO
O
O
HO
N=N
N
C14H29

**4d**

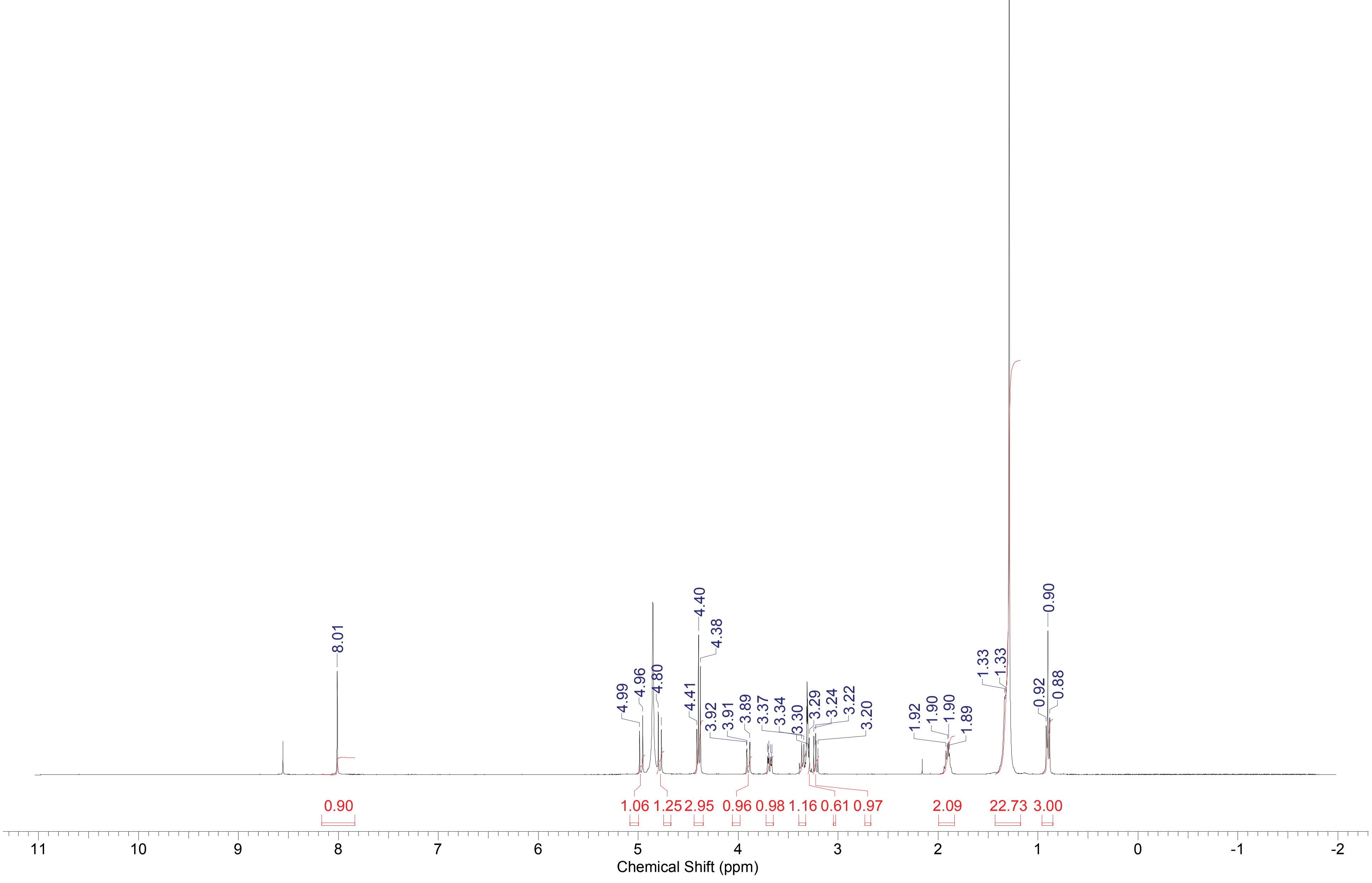
1.29
8.01
4.99
4.96
4.80
4.41
4.40
4.38
3.92
3.91
3.89
3.37
3.34
3.30
3.29
3.24
3.22
3.20
1.92
1.90
1.90
1.89
1.33
1.33
0.92
0.90
0.88
0.90
1.06
1.25
2.95
0.96
0.98
1.16
0.61
0.97
2.09
22.73
3.00
11
10
9
8
7
6
5
4
3
2
1
0
-1
-2
Chemical Shift (ppm)

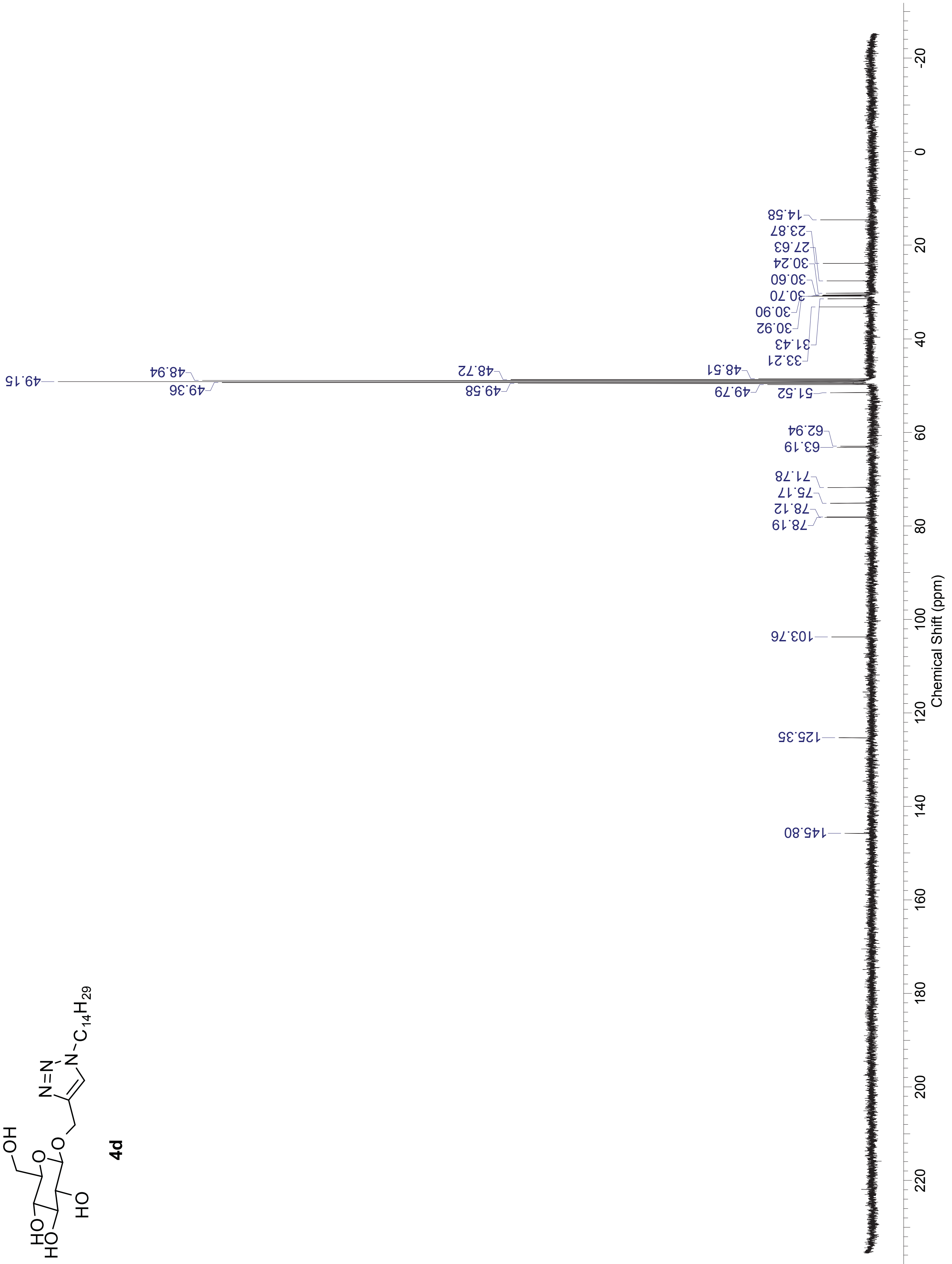

14.58
23.87
27.63
30.24
30.60
30.70
30.90
30.92
31.43
33.21
48.51
48.72
48.94
49.15
49.36
49.58
49.79
51.52
62.94
63.19
71.78
75.17
78.12
78.19
103.76
125.35
145.80
Chemical Shift (ppm)
N=N
N
$C_{14}H_{29}$
HO
OH


4d

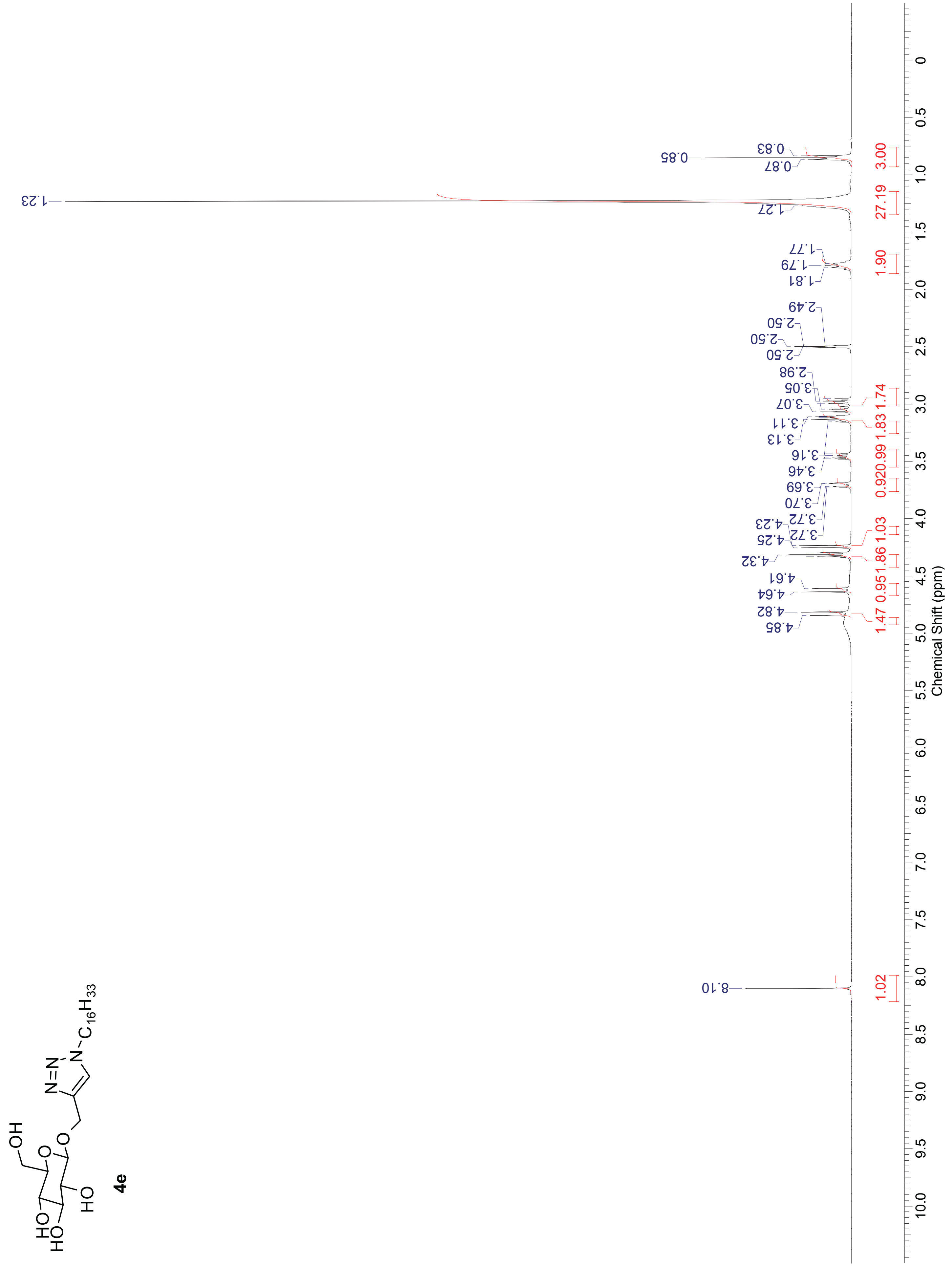

N=N
N-C16H33
OH
HO
HO
HO
O
O
Chemical Shift (ppm)
0.83
0.85
0.87
1.23
1.27
1.77
1.79
1.81
2.49
2.50
2.50
2.50
2.98
3.05
3.07
3.11
3.13
3.16
3.46
3.69
3.70
3.72
3.72
4.23
4.25
4.32
4.61
4.64
4.82
4.85
8.10
3.00
27.19
1.90
1.74
1.83
0.99
0.92
1.03
1.86
0.95
1.47
1.02


**4e**

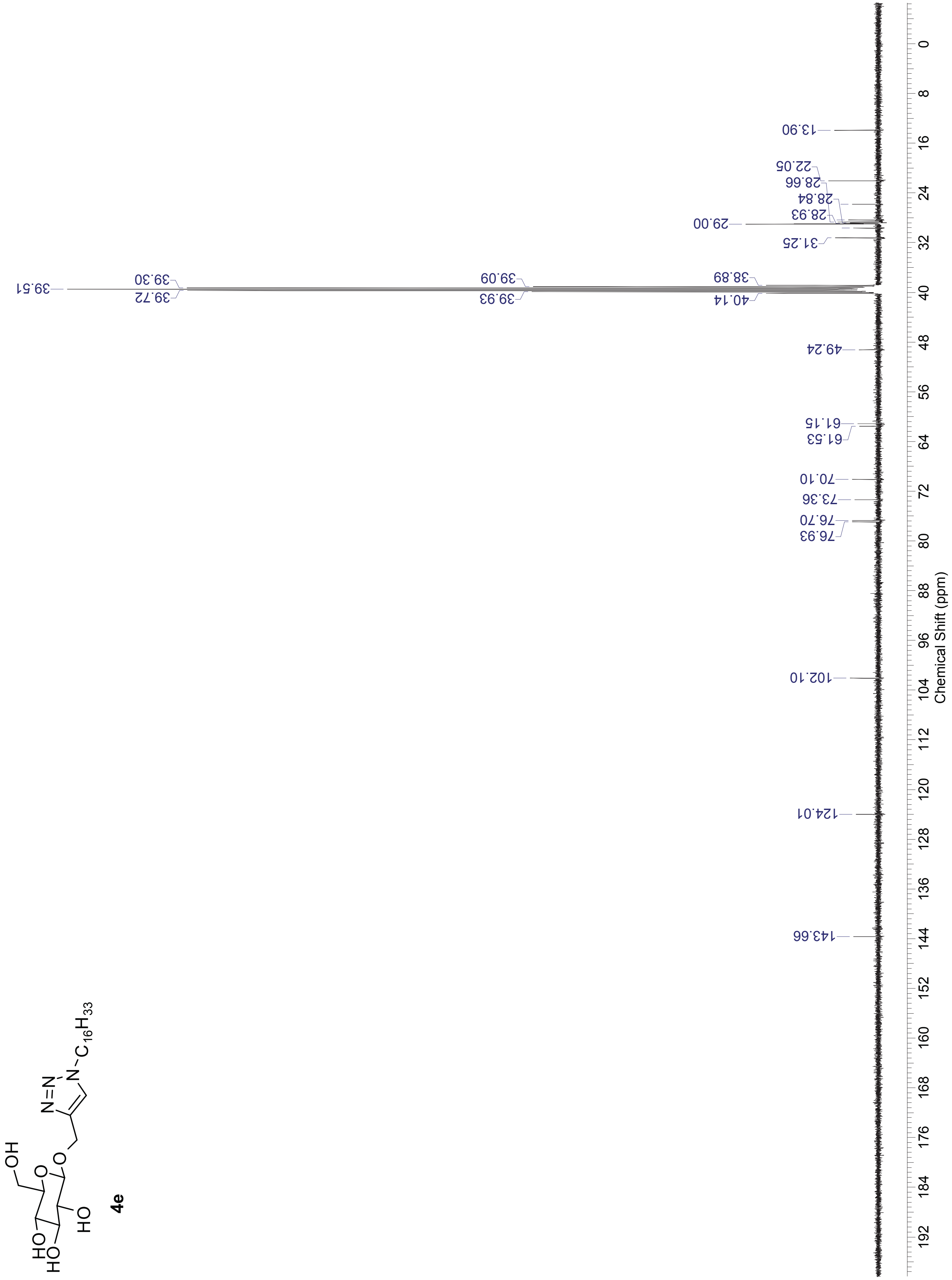
Chemical Shift (ppm)
13.90
22.05
28.66
28.84
28.93
29.00
31.25
38.89
39.09
39.30
39.51
39.72
39.93
40.14
49.24
61.15
61.53
70.10
73.36
76.70
76.93
102.10
124.01
143.66
N=N
N
C16H33
OH
HO
HO
OH
O
O

4e

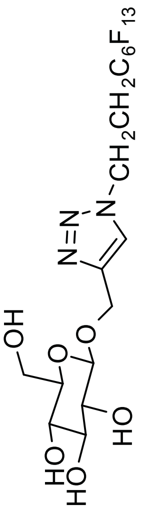
HO
HO
HO
OH
O
O
N=N
N-CH2CH2C6F13

**4f**

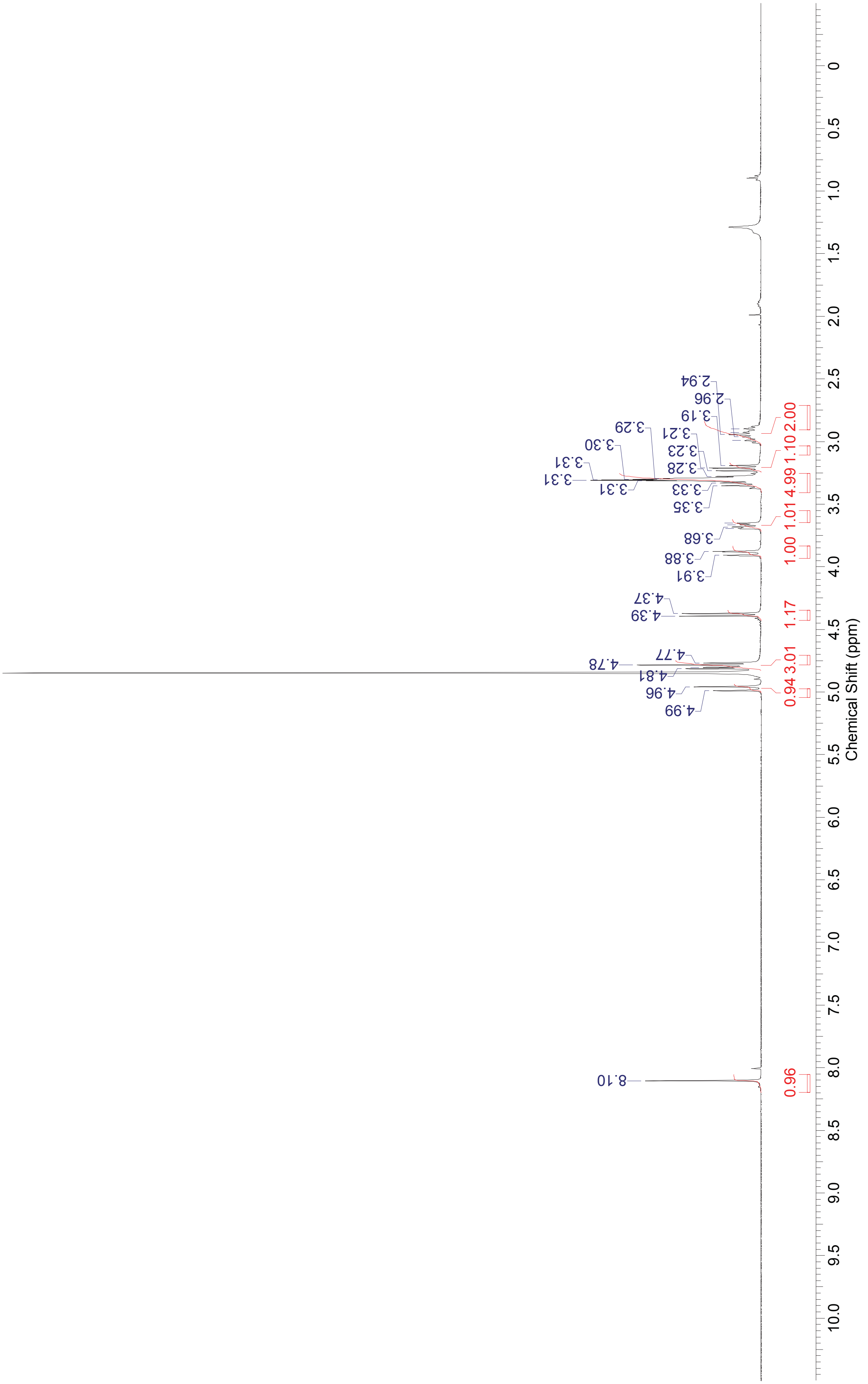
8.10
4.99
4.96
4.81
4.78
4.77
4.39
4.37
3.91
3.88
3.68
3.35
3.33
3.31
3.31
3.31
3.30
3.29
3.28
3.23
3.21
3.19
2.96
2.94
0.96
0.94
3.01
1.17
1.00
1.01
4.99
1.10
2.00
Chemical Shift (ppm)

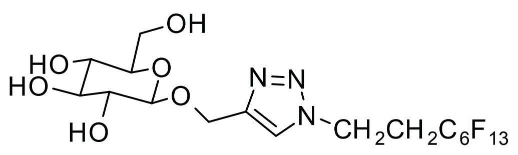
OH
HO
O
HO
O
N=N
N
CH2CH2C6F13
HO

**4f**

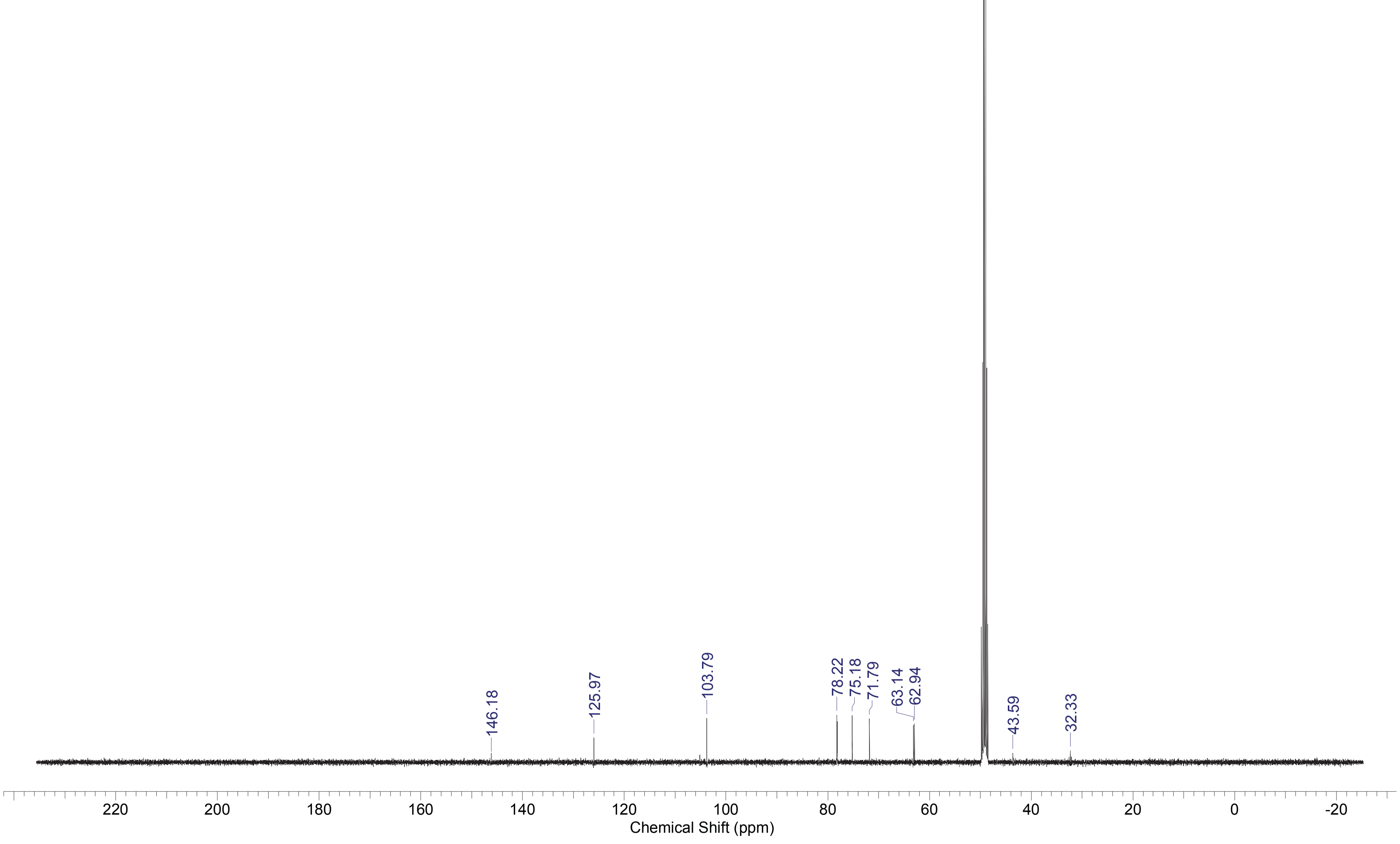
146.18
125.97
103.79
78.22
75.18
71.79
63.14
62.94
43.59
32.33
220
200
180
160
140
120
100
80
60
40
20
0
-20
Chemical Shift (ppm)

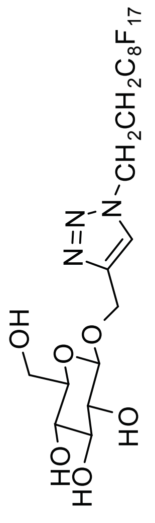

**4g**

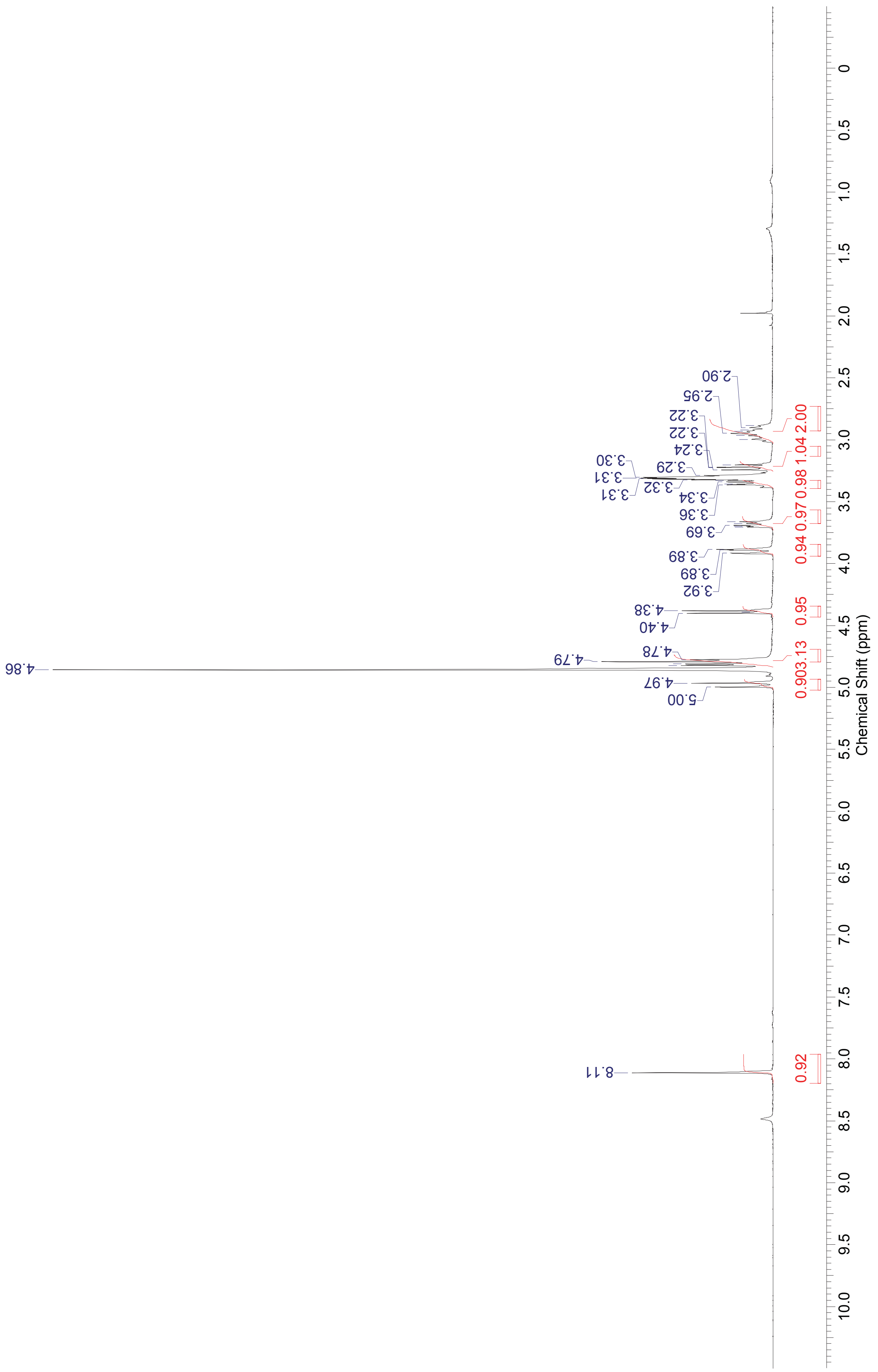

8.11
5.00
4.97
4.86
4.79
4.78
4.40
4.38
3.92
3.89
3.89
3.69
3.36
3.34
3.32
3.31
3.31
3.30
3.29
3.24
3.22
3.22
2.95
2.90
0.92
0.90
3.13
0.95
0.94
0.97
0.98
1.04
2.00
Chemical Shift (ppm)

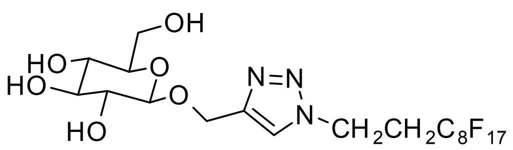

**4g**

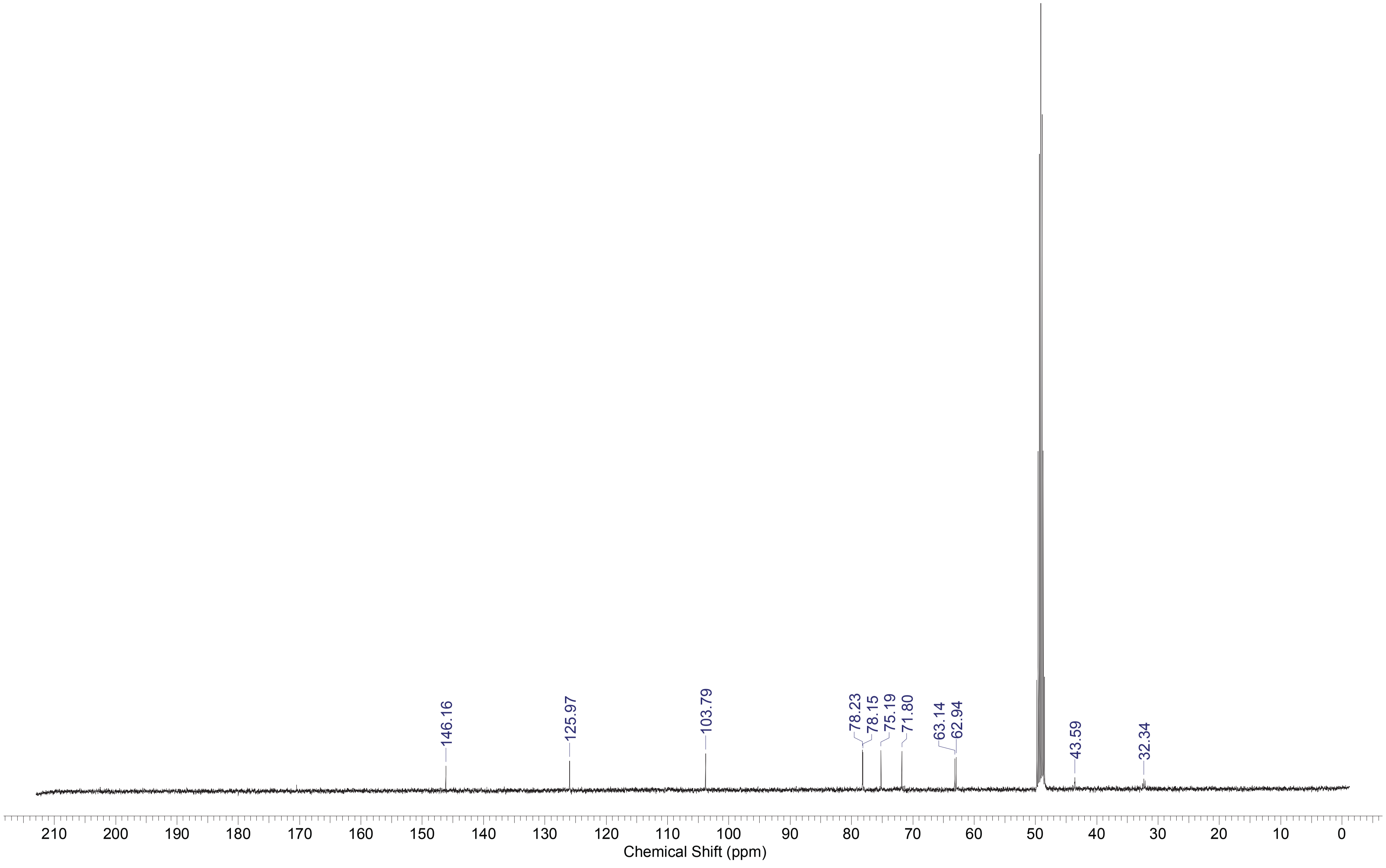

146.16
125.97
103.79
78.23
78.15
75.19
71.80
63.14
62.94
43.59
32.34
210 200 190 180 170 160 150 140 130 120 110 100 90 80 70 60 50 40 30 20 10 0
Chemical Shift (ppm)
